# WWP2 Regulates Renal Fibrosis and the Metabolic Reprogramming of Profibrotic Myofibroblasts

**DOI:** 10.1681/ASN.0000000000000328

**Published:** 2024-03-19

**Authors:** Huimei Chen, Ran You, Jing Guo, Wei Zhou, Gabriel Chew, Nithya Devapragash, Jui Zhi Loh, Loreto Gesualdo, Yanwei Li, Yuteng Jiang, Elisabeth Li Sa Tan, Shuang Chen, Paola Pontrelli, Francesco Pesce, Jacques Behmoaras, Aihua Zhang, Enrico Petretto

**Affiliations:** 1 Programme in Cardiovascular and Metabolic Disorders (CVMD) and Centre for Computational Biology (CCB), Duke-NUS Medical School, Singapore; 2 Department of Nephrology, Children's Hospital of Nanjing Medical University, Nanjing, China; 3 Nephrology, Dialysis and Transplantation Unit, Department of Precision and Regenerative Medicine and Ionian Area (DiMePRe-J), University of Bari Aldo Moro, Bari, Italy; 4 School of Science, Institute for Big Data and Artificial Intelligence in Medicine, China Pharmaceutical University, Nanjing, China; 5 Division of Renal Medicine, Fatebenefratelli Isola Tiberina—Gemelli Isola, Rome, Italy; 6 Centre for Inflammatory Disease, Imperial College London, Hammersmith Hospital, London, United Kingdom

**Keywords:** CKD, fibroblast, fibrosis, metabolism, renal fibrosis

## Abstract

**Key Points:**

- WWP2 expression is elevated in the tubulointerstitium of fibrotic kidneys and contributes to CKD pathogenesis and progression.
- WWP2 uncouples the profibrotic activation and cell proliferation in renal myofibroblasts.
- WWP2 controls mitochondrial respiration in renal myofibroblasts through the metabolic regulator peroxisome proliferator-activated receptor gamma coactivator 1-alpha.

**Background:**

Renal fibrosis is a common pathologic end point in CKD that is challenging to reverse, and myofibroblasts are responsible for the accumulation of a fibrillar collagen–rich extracellular matrix. Recent studies have unveiled myofibroblasts' diversity in proliferative and fibrotic characteristics, which are linked to different metabolic states. We previously demonstrated the regulation of extracellular matrix genes and tissue fibrosis by WWP2, a multifunctional E3 ubiquitin–protein ligase. Here, we investigate WWP2 in renal fibrosis and in the metabolic reprograming of myofibroblasts in CKD.

**Methods:**

We used kidney samples from patients with CKD and *WWP2*-null kidney disease mice models and leveraged single-cell RNA sequencing analysis to detail the cell-specific regulation of WWP2 in fibrotic kidneys. Experiments in primary cultured myofibroblasts by bulk-RNA sequencing, chromatin immunoprecipitation sequencing, metabolomics, and cellular metabolism assays were used to study the metabolic regulation of WWP2 and its downstream signaling.

**Results:**

The tubulointerstitial expression of WWP2 was associated with fibrotic progression in patients with CKD and in murine kidney disease models. WWP2 deficiency promoted myofibroblast proliferation and halted profibrotic activation, reducing the severity of renal fibrosis *in vivo*. In renal myofibroblasts, WWP2 deficiency increased fatty acid oxidation and activated the pentose phosphate pathway, boosting mitochondrial respiration at the expense of glycolysis. WWP2 suppressed the transcription of peroxisome proliferator-activated receptor gamma coactivator 1-alpha (PGC-1α), a metabolic mediator of fibrotic response, and pharmacologic inhibition of PGC-1*α* partially abrogated the protective effects of WWP2 deficiency on myofibroblasts.

**Conclusions:**

WWP2 regulates the metabolic reprogramming of profibrotic myofibroblasts by a WWP2-PGC-1*α* axis, and WWP2 deficiency protects against renal fibrosis in CKD.

## Visual Abstract

**Figure:**
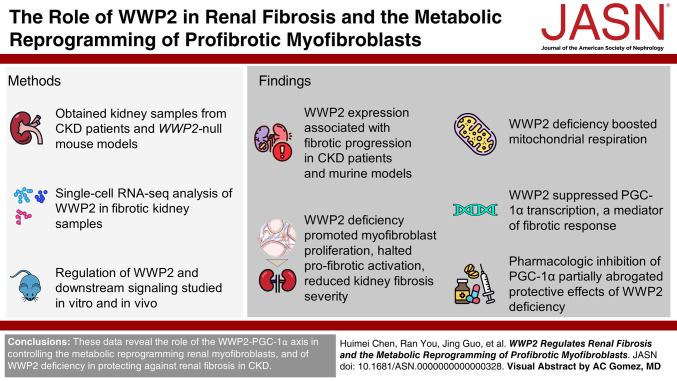

## Introduction

CKD is a global health concern and one of the leading causes of mortality worldwide. Renal fibrosis is a common pathologic pathway in CKD, irrespective of the underlying etiology.^1^ It is broadly characterized by the accumulation of a fibrillar collagen–rich extracellular matrix (ECM) in the tubulointerstitial space, which eventually results in kidney failure,^2^ and myofibroblasts are considered the main source of ECM production. Recent studies highlight the complexity of this process^3,4^ and show extensive fibroblast heterogeneity in the fibrotic (or inflamed) kidney.^5^ Alpha-smooth muscle actin (*α*-SMA) is commonly used as a specific marker for activated myofibroblasts,^6,7^ but only approximately 50% of *α*-SMA^+^ cells express ECM protein, Col1a1 (collagen type I *α* 1).^8^ Fibroblast to myofibroblast differentiation has consequences on the proliferative status of the cell,^9,10^ and this contributes to cell activation and ECM production.^11^ However, other studies showed that cell proliferation is independent of myofibroblast activation and even protective from fibrosis.^12,13^

Fibrometabolism is an emerging avenue of research,^14^ and cellular metabolic reprogramming is considered as a fundamental process for fibrosis in different organs.^5,15^ In skin wound healing, disruptions of the enzymes involved in glycolysis and the fatty acid oxidation display their reciprocal effects on ECM upregulation and downregulation, respectively.^16^ Increased aerobic glycolysis represents a critical factor for myofibroblast activation in lung fibrosis, particularly when coupled with profibrotic TGF-*β* signaling activation.^17,18^ Suppression of myofibroblast proliferation is also linked to a metabolic flux rerouting from the pentose phosphate pathway toward glycolysis, coupled with an increased expression of ECM proteins,^19^ and a reduction in the generation of NADPH has been observed in pulmonary myofibroblasts.^20^ However, considerable controversy remains regarding the functional heterogeneity of myofibroblasts and their metabolic regulation in CKD.

Altered metabolic activities have been observed in injured kidneys, often in association with fibrotic progression in CKD.^21^ Enhancing fatty acid oxidation and oxidative phosphorylation (OXPHOS) can effectively improve kidney function,^22^ while ramping down glycolysis can slow down the progression of fibrosis.^23^ Dysregulated myofibroblast metabolism may be critical for shaping cellular proliferation and profibrotic response during renal fibrosis,^24^ yet much emphasis has been given to metabolism programming in proximal tubule epithelial cells.^25–27^ The contribution of myofibroblasts metabolic reprogramming to fibrosis remains poorly defined, partly due to the high myofibroblast heterogeneity in fibrotic kidneys.

We previously identified the homologous to E6AP C-terminus domain E3 ubiquitin–protein ligase WWP2 as a regulator of myofibroblast profibrotic activation in cardiac fibrosis.^28^ Giving the widespread tissue expression and master regulatory role of WWP2 in orchestrating profibrotic ECM network,^28,29^ we hypothesized that WWP2 regulates myofibroblast phenotypes and tissue fibrosis in kidney disease. To test this hypothesis, we investigated human CKD kidneys, developed cellular and mouse models, and integrated single-cell transcriptomics with metabolic analyses to characterize the function of WWP2 in renal fibrosis. Through these studies, we propose that WWP2 underlies the metabolic reprogramming and profibrotic response of myofibroblasts during CKD. We provide evidence that these effects are in part mediated by peroxisome proliferator-activated receptor gamma coactivator 1-alpha (PGC-1α).

## Methods

### Human Samples

The Bari cohort was enrolled from the Nephrology Unit of the University of Bari, Italy. The protocol was approved by the Independent Ethics Committee of the “Azienda Ospedaliero Universitaria Policlinico Consorziale di Bari” and was conducted in accordance with the Declaration of Helsinki (Prot. N.4104/2013). In the Bari cohort, we analyzed 34 patients with a histologic diagnosis of FSGS, 37 patients showing evidence of IgA nephropathy, and 39 patients with membranous nephropathy. The Nanjing cohort was enrolled from the Department of Nephrology in the Children's Hospital of Nanjing Medical University in Nanjing, China. The protocol was approved by the Independent Ethics Committee of the hospital and was conducted in accordance with the Declaration of Helsinki (No. 202304069-1). In the Nanjing cohort, we analyzed 23 patients diagnosed with IgA nephropathy.

All patients from both cohorts gave written informed consent for the use of biopsy material for research purposes. Detailed information on those patients is presented in Supplemental Table 1. Biopsy specimens were evaluated through light, immunofluorescence, and electron microscopy independently by two nephropathologists, blinded to patients' features to diagnose the disease.

### Mouse Models

Mice were bred and maintained in the animal facility of Duke-NUS Medical School before use. Protocol with Institutional Animal Care and Use Committee number 2021/SHS/1653 was approved by Institutional Animal Care and Use Committee of the National University of Singapore, Duke-NUS Medical School, Singapore. All mice were housed in a specific pathogen-free environment and complied with all relevant ethical regulations according to guidelines issued by the National Advisory Committee on Laboratory Animal Research. The housing room was set to a 12-hour light/dark cycle with lights off at 8 am, a temperature of about 22°C, and a relative air humidity of about 50%. Steps were performed to minimize animal suffering according to guidelines of the SingHealth Council on Animal Care.

With respect to the unilateral ureteral obstruction (UUO) model, mice underwent ligation of the left ureter and were sacrificed on day 7 or day 14. For the folic acid model, mice were injected with folic acid (250 mg/kg once, dissolved in 300 mM NaHCO_3_) intraperitoneally and sacrificed on day 21.

### Mouse Strain

WWP2^−/−^ mouse line was previously generated in our laboratory as described,^28^ and littermates were used from in-house mating in the vivarium at Duke-NUS Medical School, Singapore. The background of mice is C57BL/6J (B6J). Pairs of female and male heterozygous WWP2^+/−^ mice were housed together for breeding. Homozygous WWP2^−/−^ and wild-type (WT) litter mates were weaned at around 3 weeks of age and housed in same-sex groups of four to five animals per cage. Mice were randomly assigned to experimental test and control groups.

Transgenic WWP2 mice (WWP2^Tg^) overexpressed mouse WWP2 full-length isoform under the immediate-early promoter of cytomegalovirus on the pIRES2-EGFP Vector. In detail, 2 ml plasmid saline solution was administered to mice *via* tail vein injection at the dosage of 4 mg/kg within 2 seconds. Mice of 8 weeks were randomly assigned to ControlTg and WWP2^Tg^ undergoing plasmid injection containing control and WWP2 DNA insert, respectively. The efficiency of WWP2 overexpression in the kidneys was examined 24 hours after injection by randomly selecting three mice from each group; the UUO modeling was established 24 hours after injection in the rest of the mice.

### Primary Myofibroblasts Culture

Murine renal (myo)fibroblasts were derived from mouse kidneys using the primary culture of kidney tissue pieces.^30,31^ In detail, minced kidney pieces (1–3 mm^3^) were placed in 6-cm dishes with Dulbecco's Modified Eagle Medium (DMEM) supplemented with 20% fetal bovine serum. The fibroblasts began to crawl out of the tissues at approximately 5 days and reached to 80%–90% confluence around tissue pieces at approximately 10 days. These cells were labeled as P0 and passaged 1–2 times (P1–P2) for experiments in DMEM supplemented with 10% fetal bovine serum. P2 renal myofibroblasts (at approximately 80% confluence) were used for all experiments. All cells were cultured and passaged in parallel, and in each experimental batch, P1–P2 cells were treated simultaneously.

WWP2^OE^ cells were generated with infection of lentivirus, which carried antipuromycin gene and WWP2 full-length isoform DNA, and the lentivirus with scramble DNA as control. Seventy-two hours after infection, 4 *μ*g/ml puromycin was added in the medium to deplete the noninfected cells. To activate myofibroblasts, cells were treated with TGF*β*1 human at a concentration of 5 ng/*μ*l for 24 or 72 hours, as indicated in each experiment. To inhibit or activate PGC-1*α* function, cells were treated with 10–20 μM ZLN005 and SR18292 at 2.5–10 *μ*M for 30 minutes before TGF*β*1 treatment for 72 hours, respectively.

### Kidney Histopathology

Masson's trichrome and Sirius Red were performed on paraffin-embedded sections to quantify the percentage of fibrotic area in kidneys. Images were acquired by the Aperio ScanScope CS2 device (Aperio Technologies, Vista, CA), and digital slides were analyzed by ImageScope V12.1.0.5029 (Aperio Technologies). We excluded for the analysis perivascular regions, Bowman's capsule, and the limits of each biopsy section were defined by drawing tools. The positive area was expressed as the ratio positive area over the total area analyzed (%).

### Hydroxyproline Assay

The amount of total collagen in the kidneys was quantified using the Quickzyme Total Collagen assay kit (Quickzyme Biosciences). The assays were performed according to the manufacturer's protocol. The levels of hydroxyproline assay collagen were normalized to the weight of kidneys tested and expressed as *μ*g/mg.

### Immunochemistry and Immunofluorescence Staining

For the immunohistochemical evaluation of WWP2 in human kidneys, 4 *μ*m thick paraffin-embedded kidney tissues underwent deparaffination and heat-mediated antigen retrieval (EDTA 1 mM, pH=8.00). After epitope unmasking, the slides were incubated with H_2_O_2_ (0.3%) and then with Triton X (0.25%), protein block solution (Dako), and the primary antibody (1:50 dilution in PBS). Primary antibodies were detected by the Peroxidase/DAB Dako Real EnVision Detection System according to the manufacturer's instructions (Dako). Kidney sections were counterstained with Mayer hematoxylin and mounted with glycerol (Dako Cytomation, Glostrup, Denmark). Negative controls were prepared using isotype control antibodies.

For the immunofluorescence staining of kidney samples, sections of 5 μm thickness of kidneys were fixed with 10% neutral buffered formalin and processed using Leica automatic tissue processor. Following dewaxing and rehydration, sections were heated in citrate buffer for antigen retrieval for further antibody incubation. For cell sections, P2 renal fibroblasts were seeded onto eight well removable chamber slides (80841, ibidi) and fixed with ice-cold acetone for 30 minutes at room temperature. Antibodies used for immunofluorescence staining are as follows: anti-ACTA2 (Sigma-Aldrich, 1:100), anti-WWP2 (Bethyl Laboratories, 1:100), and anti-Vimentin (Abcam, 1:100). Images were visualized using secondary antibodies at 1:500 for 2 hours at room temperature and imaged under a Leica Fluorescent microscope.

### Oil Red O Staining

Renal myofibroblasts were seeded onto 24-well plate at a density of 8000 cells/well. After treatment with TGF*β*1 (5 ng/*μ*l) for 72 hours, cells were fixed with 4% paraformaldehyde for 30 minutes at room temperature. After brief rinsing with PBS, plates were incubated with Oil Red O solution (0.5% in isopropanol, O1391, Sigma-Aldrich) for 20 minutes at room temperature. Images were obtained using a light microscope after rinsing the plates with dH_2_O. Quantification of Oil Red O stain extracted from stained cells was performed by eluting the stain from cells in 1 ml of 100% isopropyl alcohol and then measuring the absorbance of the stain against a blank (100% isopropyl alcohol) at 500 nm.

### Quantitative Real-Time PCR Analysis

Total RNA was extracted from snap-frozen tissue and primary cells using the RNeasy mini kit (Qiagen), and cDNA was prepared using iScript cDNA synthesis kit (primer specific, Biorad) according to the manufacturer's instructions. Fast SYBR-Green master mix (Biorad) was used for the analysis of gene expression using the Biorad CFX reverse transcription-PCR system. The primers used in the experiment are listed in Supplemental Dataset 1. 18S was used to normalize the relative gene expression, and 2^−ΔΔCt^ method was used to measure the gene expression changes.

### Coimmunoprecipitation

To detect WWP2 and TWIST1 proteins interaction, the lysates obtained from the renal myofibroblasts (approximately 5×10^6^ each sample) were immunoprecipitated with the anti-WWP2 (Bethyl Laboratories) following the manufacturer's instructions using Pierce Direct Magnetic IP/CO-IP kit (88828, Thermo fisher). Rabbit IgG (AS21 4694) served as a negative immunoprecipitation control.

### Western Blot Analysis

Cell lysates were obtained from kidney tissues and cells using radioimmunoprecipitation assay buffer (Thermo Fisher Scientific) supplemented with protease (Sigma-Aldrich) and phosphatase inhibitor cocktails (Roche). Lysates were subjected to 4%–12% SDS-PAGE electrophoresis after Bradford quantification. Blotting of the membrane was performed using anti-WWP2 (Bethyl Laboratories, 1:250), anti-ACTA2 (Sigma-Aldrich, 1:500), anti-Vimentin (Abcam, 1:500), anti-Periostin (Novus Bio, 1:500), anti-Fibronectin (Sigma-Aldrich, 1:500), anti-Col1a1 (SouthernBiotech, 1:500), anti-PGC1a (Abcam, 1:500), anti-CyclinA (GeneTex, 1:500), and anti p21 (GeneTe, 1:500) after transfer onto a nitrocellulose membrane at 100 V for 1 hour. Antitubulin (Sigma-Aldrich, 1:5000) and anti-glyceraldehyde 3-phosphate dehydrogenase (Abcam, 1:5000) were used as loading controls. Full and unprocessed scanned images of the blots are provided in Supplemental Dataset 2.

### Flow Cytometry Analysis

Mouse kidneys were perfused, excised, minced, and digested with Collagenase II (Worthington Biochemical Corporation) and Dispase II (Roche). Tissue mixture was mechanically disrupted and filtered through 70 *μ*m cell strainer to obtain single-cell suspension. Cultured myofibroblasts were seeded onto 10-cm dishes and were harvested for single-cell suspension after treatment with TGF*β*1 (5 ng/*μ*l) for 72 hours. After blocking with CD16/32 (Thermo fisher, 1:100) at reverse transcription for 10 minutes, cells were fixed and permeabilizated using Intracellular Fixation and Permeabilization Buffer Set (eBioscience). Cells were collected by centrifugation, subjected to live-death dye and antibody staining, and analyzed (or sorted) by flow cytometry using BD FACS ARIA system; the data were analyzed using FlowJo v10 software.

### Cell Proliferation Assay

Cell proliferation was assessed using the 3-(4,5-dimethylthiazol-2-yl)-5-(3-carboxymethoxyphenyl)-2-(4-sulfophenyl)-2H-tetrazolium (MTS) assay (Promega) according to the manufacturers' protocol. For the cell cycle assay, cells were harvested and fixed using the Propidium Iodide Flow cytometry Kit (Abcam) and measured using the BD FACS ARIA system (BD Bioscience, Franklin Lakes, NJ). Data were analyzed using ModFit LT software (DNA Modeling System).

### ChIP-Seq and Chromatin Immunoprecipitation Quantitative PCR Analyses

Chromatin immuno-precipitation (ChIP) assay was performed using Magnetic ChIP Kit (Pierce) with anti-WWP2 antibody (A302-936A, Bethyl Laboratories). In brief, P2 WT renal fibroblasts were grown for 80%–90% confluence. After treatment with TGF*β*1 (5 ng/*μ*l) for 24 hours, cells were harvested by trypsinisation. DNA and proteins in the cells (approximately 10^6^ in each sample) were cross-linked using 1% (v/v) formaldehyde and then sonicated in lysis buffer to obtain 200–500 bp long DNA fragments. Supernatant was incubated with control IgG or WWP2 antibody beads at 4°C or kept as input reference. Reverse-crosslinking and purification of DNA was performed.

Chromatin immunoprecipitation sequencing (ChIP-seq) libraries were constructed and sequenced using Illumina HiSeq 4000 sequencer, resulting in paired-end FASTQ files containing sequence reads of 150 bp length, which were mapped to mouse reference genome GRCm38 (mm10) v89 using STAR aligner. Peak calling was performed by MACS2 callpeak function^32^ using the following parameters: “-qvalue 0.001,” “–keep-dup auto,” and “–call-summits.” The genomic location of the peak maximum was extended 250 bp both upstream and downstream, defining a 501 bp–long sequence centered on each ChIP-seq peak. Transcription factor binding site (TFBS) motifs discovery and annotation was performed by the Regulatory Sequence Analysis Tools (RSAT) peak-motif web server^33^ (with default parameters) and using the JASPAR (an open access database of curated, nonredundant TFBS profiles)^34^ core nonredundant vertebrates and ENCODE databases.^35^ The results of all the TFBS motifs identified are presented in Supplemental Table 2. The correlations of TFBS motifs with DNA sequences underlying ChIP-Seq–binding peaks were obtained by RSAT.

### Bulk RNA-Seq Data Generation and Analysis

Primary renal myofibroblasts (P2) were harvested, and RNA was extracted using the RNeasy mini kit (Qiagen) following the manufacturer's instruction. Libraries were generated with the poly-A selection method (mRNA Direct kit, Life Technologies) and sequenced using the NovaSeq 6000 S4 platform (2×150 bp) with a target of 30 million reads per library. Libraries were sequenced using paired-end 150 bp sequencing strategy. Reads were mapped to the mouse genome GRCm38 (mm10) v89 using STAR aligner,^36^ and counts per gene were quantified by featureCounts^37^ with settings of pair-end (-p) and exon mapping (-t exon). Genes were considered detected if they have at least five read counts in at least five samples. Counts were then normalized for sequencing depth and RNA composition across all samples by DESeq2.^38^

Differential gene expression analysis between two sample groups (WT and WWP2^−/−^ mouse) was performed by the Wald test, the default test in DESeq2. After multiple testing correction, genes with a false discovery rate <0.05 were considered significantly differentially expressed, unless otherwise indicated. Functional enrichment analysis of the differentially expressed genes between WT and WWP2^−/−^ was performed by clusterProfiler, and visualization of enrichment results was performed by gseaplot function. Pathways relevant to ECM and cell proliferation were identified using curated gene sets from Molecular Signatures Database.^39,40^

### Metabolic Function Analysis

Metabolic measurements of myofibroblasts were obtained by real-time oxygen consumption rate and extracellular acidification rate (ECAR) using a Seahorse XFe96 Analyzer (Agilent). In brief, 10,000–20,000 cells were seeded into each well of a XF96 cell culture microplate (102416-100, Agilent). Cells were isolated from one mouse and then plated on 3–4 microplate wells; experiments were performed independently three times (*i.e*., using three different mice). For Cell Mito Stress Test analysis, the culture medium was switched to Seahorse XF DMEM assay medium with 1 mM pyruvate, 2 mM glutamine, and 10 mM glucose and then placed into a CO_2_ free 37°C incubator for 1 hour before the assay. The oxygen consumption rate measurements were recorded using serial injections of Oligomycin (10 *μ*m), fluoro-carbonyl cyanide phenylhydrazone (1 *μ*M), and antimycin A (0.5 *μ*M) plus rotenone (0.5 *μ*M). For the Glycolysis Stress Test analysis, the culture medium was changed to Seahorse XF DMEM supplemented with 2 mM glutamine and then placed into a CO_2_ free 37°C incubator for 1 hour before the assay. The ECAR measurements were recorded using serial injections of glucose (15 mM), Oligomycin (10 *μ*m), and 2-deoxy-D-glucose (50 mM). On completion of each Seahorse XF assay, the medium was discarded, and the cells were incubated in 0.5 mg/ml MTS for 3 hours for normalization. The number of cells per well was normalized by MTS assay, which was used to measure cellular metabolic activity.

### Metabolomics Analysis

After treatment with TGF*β*1 (5 ng/*μ*l) for 72 hours, primary renal myofibroblasts (P2) were harvested in 0.6% formic acid and sonicated with ten cycles (30 seconds ON, 30 seconds OFF). Acetonitrile was added in a 1:1 ratio to the formic acid volume, and the mixture was vortexed for homogeneity. We used a metabolomics approach to quantify acetylcarnitines, amino acids, glucose molecules, and organic acids involved in fatty acid oxidation, amino acid metabolites, glycolysis, and TCA cycle intermediates, which was conducted in the Metabolomics Facility at Duke-NUS Medical School, Singapore.

The glycolytic intermediates were separated by capillary ion chromatography on a Dionex ICS-4000 capillary system (Thermo Fisher Scientific) and monitored on a Thermo Q Exactive Plus Q-Orbitrap high-resolution mass spectrometry (Thermo Fisher Scientific). For amino acids and acylcarnitine (AC) panel, the samples were run with a C18 column (Phenomenex, 100×2.1 mm, 1.6 *μ*m, Luna Omega) on 1290 Infinity LC system (Agilent Technologies) coupled with quadrupole-ion trap mass spectrometer (QTRAP 5500, AB Sciex). Trimethylsilyl derivatives of organic acids were separated by gas chromatography (Agilent Technologies, HP 7890A) and quantified by selected ion monitoring on a 5975C mass spectrometer using stable isotope dilution. Data acquisition and analysis were performed on MassHunter Workstation B.06.00 (Agilent), MultiQuant 3.0.3 software (AB Sciex, DC), and using the TraceFinder software (Thermo Fisher Scientific). Data were normalized with respect to the protein concentration. Statistical differences between WT and WWP2^−/−^ groups were assessed by two-tailed nonparametric Mann–Whitney *U* test, and *P* values were corrected using the Benjamini–Hochberg (BH) method.

### Single-Cell RNA-Seq Data Generation and Analysis

#### Library Generation and Sequencing

Mouse kidneys were perfused, excised, minced, and digested to obtain single-cell suspensions as described above. Cells were collected by centrifugation, subjected to live-death dye, and sorted by flow cytometry using BD FACS ARIA system (BD Bioscience, Franklin Lakes, NJ). Isolated living single-cell suspensions were converted to barcoded single-cell RNA sequencing (scRNA-seq) libraries by using the Chromium Single Cell 3′ Library, Gel Bead and Multiplex Kit, and Chip Kit V3, loading an estimated 7000–12,000 cells per library/well, and following the manufacturer's instructions. Indexed libraries were sequenced using Illumina NovaSeq 6000 platform, where 150 bp pair-end sequences were obtained. Sequencing reads were aligned and quantified to the mouse genome GRCm38 (mm10–3.0.0 provided by 10× Genomics) using 10× Genomics Cell Ranger count.

#### Data Preprocessing and Cell Annotation

Quality control and preprocessing were performed individually for each of the four samples (WWP2^−/−^, *n*=2 and WT, *n*=2). Genes detected in <50 cells and cells below the tenth and above the 90th percentile in terms of both the number of genes and number of counts were filtered out. Cells with percentage of mitochondrial content >40% and hemoglobin gene accounting more than 1% of total counts were also filtered out. In addition, cells above the 99th percentile in terms of percentage of total counts being stress genes^41^ were also removed. Seurat (v4.0) was used to normalize the filtered data using normalization method of “LogNormalize” and scale factor of 10,000. The top 5000 highly variable genes were used for the principal component analysis, and top 50 principal components were used for Uniform Manifold Approximation and Projection dimensionality reduction. Doublet identification and the calculation of doublet score were performed using coexpression-based doublet scoring.^42^ Clusters with median doublet score of greater than the 60th percentile were denoted as potential doublet clusters and filtered out. Small outlier clusters with fewer than 1% of total number of cells were also filtered out. For cell type annotation, markers from a published scRNA-seq study of murine kidney using the UUO model^43^ were used as reference. Cell type scores were calculated using Seurat (v4.0) AddModuleScore function with random seed set at 42 and control gene set size of 500. Final cell type annotations were obtained by identifying the cell type with the highest normalized score (dividing each cell type score by the maximum cell type score). The cell type annotations were further confirmed by using two other scRNA-seq datasets from murine kidney.^44,45^ Renormalization of the filtered data was performed using Seurat (v4.0) with the clustering resolution of 0.8. We recalculated doublet score using the coexpression-based doublet scoring approach as above. Clusters with median doublet score of greater than the 95th percentile were denoted as potential doublet clusters and filtered out. This yielded four processed data matrices corresponding to the initial four scRNA-seq 10× libraries. The four processed datasets were merged, and the same Seurat pipeline highlighted above was implemented using a clustering resolution of 1.0. After merging, mixed clusters were filtered out. In addition, batch-specific clusters, which have one batch dominating at least 75% of cells, were removed. This process was performed recursively until there were no batch-specific or doublet clusters present. This merged dataset was renormalized using the same Seurat (v4.0) pipeline with a clustering resolution of 1.0. Finally, small clusters (accounting for fewer than 0.25% of total cell number) and 12 outlier cells were filtered out, giving us a final processed dataset consisting of 17,058 genes and 74,585 cells. Differential gene expression analysis and functional enrichment tests: differential expression between WWP2^−/−^ and WT was performed by running Seurat's FindMarkers function with minimum log-fold change of 0 and minimum percentage set to 0. Functional enrichment test in the differentially expressed genes in fibroblast between WT and WWP2^−/−^ was performed by clusterProfiler using pathways in the Reactome database^46^ (release 83). Enriched terms were grouped by treeplot function that hierarchically clustered terms based on the pairwise similarities, which were calculated by the pairwise_termsim function. The final plot of hierarchical clustering of pathways was further manually curated by merging similar pathways/processes terms on the basis of the similarity of enriched genes. Full enrichment results are presented in Supplemental Table 3.

#### Transcription Factor Network Analysis

We derived transcriptional networks (regulons) for the mouse myofibroblasts by pyScenic standard pipeline (pySCENIC 0.11.2), using default parameters and random seed=10. This approach was used to identify regulons (*i.e*., transcription factors [TFs] and the network of the respective downstream targets) in murine renal fibroblasts. To ascertain the regulons regulated by Wwp2^−/−^, we assessed the differential expression of each regulon in WT and Wwp2^−/−^ myofibroblasts by two-sided Wilcoxon test, and the *P* value was adjusted for multiple testing using the BH method. This analysis yielded a total of 35 regulons expressed in renal fibroblasts and significantly regulated by Wwp2 (BH-adjusted *P* < 0.05), presented in Supplemental Table 4.

#### Ligand–Receptor Interaction Analysis

CellphoneDB (v2.0) was used for ligand–receptor interaction analysis in myofibroblasts and tubular cells. NicheNetR (version 1.0.0) was used to convert mouse gene symbols to human analogs, and we focused only on the myofibroblast-activating ligands among the cytokines and growth factors produced by tubular cells. Sankey flow diagram to visualize the ligand–receptor relationships was constructed using plotly (v4.9.3). Differential expression analysis of ligands and receptors between Wwp2^−/−^ and WT cells was performed using a two-sided Wilcoxon test, followed by BH *P* value adjustment for multiple testing.

#### Functional Enrichment Analysis

Gene set enrichment analysis (GSEA) and therein the clusterProflier's GSEA function was used with functional gene sets downloaded from Bader lab.^47^ Note that the normalized expression scores were weighted by the overlap between the input gene sets and the canonical functional gene sets. BH method was used for *P* value correction, and adjusted *P* value cutoff was set at 0.05 unless otherwise indicated.

#### External Datasets of Human Kidney Fibroblasts

Two human single-cell kidney datasets,^10,48^ including fibroblasts from both CKD and healthy patients' kidneys, were used in our study, and the original cell annotation provided by authors was used for downstream analysis. For the dataset “human_CD10negative_final.RData” (https://zenodo.org/record/4059315), we used fibroblasts annotated as “Fibroblast” and “Myofibroblast” cells in “Annotation.Level.3.” For the Kidney Precision Medicine Project dataset “snRNA_kidney_atlas_KPMP.rds” (https://cellxgene.cziscience.com/collections/bcb61471-2a44-4d00-a0af-ff085512674c), we used cells annotated as “kidney interstitial fibroblast” in “celltype.” For both datasets, data from patients (both healthy and CKD) with <20 fibroblasts were removed in downstream analyses.

#### Pseudotime Analysis

Slingshot was used for pseudotime analysis of cell state changes. ECM gene sets (NABA_MATRISOME, NABA_CORE_MATRISOME, NABA_BASEMENT_MEMBRANE, NABA_ECM_REGULATORS, NABA_ECM_GLYCOPROTEINS, NABA_COLLAGENS, NABA_PROTEOGLYCANS)^39^ were downloaded from GSEA Molecular Signatures Database.^49^ The ECM gene sets were integrated, and the average expression scores of ECM gene sets visualized on Uniform Manifold Approximation and Projection reduction dimension along the trajectory inferred by Slingshot.

#### Cell Phase Estimation and Visualization

The cell phase was determined for each cell using the Revelio method.^50^ This method extracts a set of marker genes for each cell phase (M–G1, G1–S, S, G2, G2–M) and, for each cell, calculates an expression score for each cell phase. The cell status was assigned based on the maximum z-score across all cell phases. Using dimensionality reduction method, all cells were projected into two-dimension on the basis of their dynamical components, which are similar to principal components. The visualization of the “pseudotime” of cell phases was the revolution of cartesian to polar transformation of the 2D dynamical component plot (more technical details^50^). The line summarizing expression score in the cell cycle plots was added by geom_smooth function, which uses a generalized additive mode smoothing method. To test if gene expression changes significantly along the cell cycle phases (G0>G1>S>G2>M), we used the WAVK trend test,^51^ which is implemented in the R package funtimes v9.1.^52^ In brief, WAVK is a nonparametric test to detect (non)monotonic parametric trends in time series; here, the time series is represented by the pseudotime trajectory alongside the cell cycle phases (G0>G1>S>G2>M), and the null hypothesis tested the by WAVK trend test is no change in trend along the pseudotime trajectory. For illustration purpose, phases M–G1 and G1–S are labeled as G0/G1, and phases G2 and G2 to M are labeled as G2/M.

**Figure 1:**
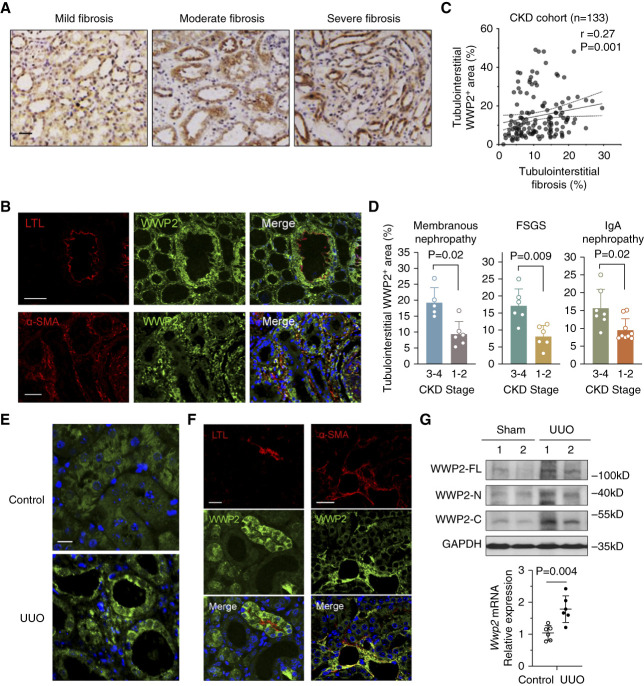
**The tubulointerstitial WWP2 expression is positively associated with renal fibrosis in CKD patients and UUO mice.** (A) Representative immunostaining images of WWP2 in tubulointerstitial area from human kidney biopsy samples, illustrating mild, moderate, and severe interstitial fibrosis, respectively (*n*=133 patients with CKD recorded, see Supplemental Table 1). Scale bars, 30 *μ*m. (B) Representative fluorescence images of WWP2 in CKD kidney. Upper panels: double-immunofluorescence staining of WWP2 (green) and lotus tetragonolobus lectin (LTL) (red). Lower panels: double-immunofluorescence staining of WWP2 (green) and *α*-SMA (red). Nuclei were stained with DAPI. Scale bars, 50 *μ*m. (C) Positive correlation between tubulointerstitial WWP2-positive area and fibrosis-positive area, as determined by immunostaining and Sirius red staining (see Supplemental Figure 1A) with ImageScope. Each data point represents a measurement of an individual section obtained from each patient with CKD (*n*=133). (D) The WWP2-positive tubulointerstitial area (%) in kidney biopsy samples increases as CKD progresses from stage 1–2 to stage 3–4, including kidney biopsy samples from patients with membranous nephropathy (*n*=11, *P* = 0.02), FSGS (*n*=12, *P* = 0.009), and IgA nephropathy (*n*=16, *P* = 0.02). *P* values calculated by the two-tailed Mann–Whitney *U* test. (E) Representative immunostaining images of WWP2 in tubulointerstitial kidneys from UUO and control mice (*n*=5–10 images recorded for each mouse kidney). Scale bars, 100 *μ*m. (F) Representative fluorescence images of WWP2 in UUO kidney. Left panels: double-immunofluorescence staining of WWP2 (green) and LTL (red). Right panels: double-immunofluorescence staining of WWP2 (green) and *α*-SMA (red). Nuclei were stained with DAPI. Scale bars, 50 *μ*m. (G) The expression of WWP2 in kidney tissue from UUO and control mice. Left: representative Western blotting for protein levels; right: mRNA expression changes were determined by RT-qPCR. *n*=6, each group, and values are reported and mean±SD. *α*-SMA, alpha-smooth-muscle actin; DAPI, 4′,6-diamidino-2-phenylindole; GAPDH, glyceraldehyde 3-phosphate dehydrogenase; RT, reverse transcription; UUO, unilateral ureteral obstruction.

#### Estimation of Cell Metabolic Activities from scRNA-Seq Data

We used the Compass algorithm^53^ to estimate the cellular metabolic states on the basis of single-cell RNA expression and infer flux balance. For each cell, compass calculates the activity scores of a comprehensive list of metabolic reactions. The differences of these activities scores between WWP2^−/−^ and WT group were tested using the nonparametric Wilcoxon rank-sum test, and the effect size was estimated by Cohen's D. BH-adjusted *P* value < 0.01 was considered as significant. For each cell, we also calculated the correlation between the *WWP2* mRNA expression (normalized counts with scale factor 10k) and the metabolic activity scores by Spearman correlation coefficient. *P* value < 0.01 was used to identify significant correlations.

#### Correlation and Coexpression Network Modules

We used the bigSCale analytical framework^54^ to estimate the gene–gene correlation coefficient (*r*) and infer the network of genes centered on PGC-1*α* (*PPARGC1A*) in the scRNA-seq myofibroblasts data from WT and WWP2^−/−^ mouse kidneys. The genes correlated (|*r*|>0.5, *P* < 0.001) with PGC-1*α* defined two gene coexpression networks, positively correlated and negatively correlated network, respectively. The genes in each network were further clustered into different submodules using multilevel modularity optimization algorithm for finding community structures, cluster_louvain (igraph package^55^ with parameter resolution=1 which yields fewer clusters), which inferred two main modules of strongly interrelated genes. In detail, modules 1 and 2 include genes positively correlated with PGC-1*α*, while modules 3 and 4 include genes negatively correlated with PGC-1*α*. For illustration purposes, genes in modules 1 and 2 or modules 3 and 4 were grouped together, and we considered only the genes with gene–gene correlations |*r*|>0.6 and strongly correlated with PGC-1*α* (*i.e*., |*r*|>0.8). Differences in module gene expression between WWP2^−/−^ and WT were estimated using bulk RNA sequencing (RNA-seq) data (see Bulk RNA-Seq Data Generation and Analysis above) generated in cultured renal fibroblasts, with or without TGF*β*1 treatment, from WT and WWP2^−/−^ mice. First, for each gene in a module, transcript per million expression was calculated and normalized by z-score across all samples. Second, for each module, global expression differences between WWP2^−/−^ and WT were tested using the nonparametric Wilcoxon rank-sum test on the z-score normalized data.

**Figure 2:**
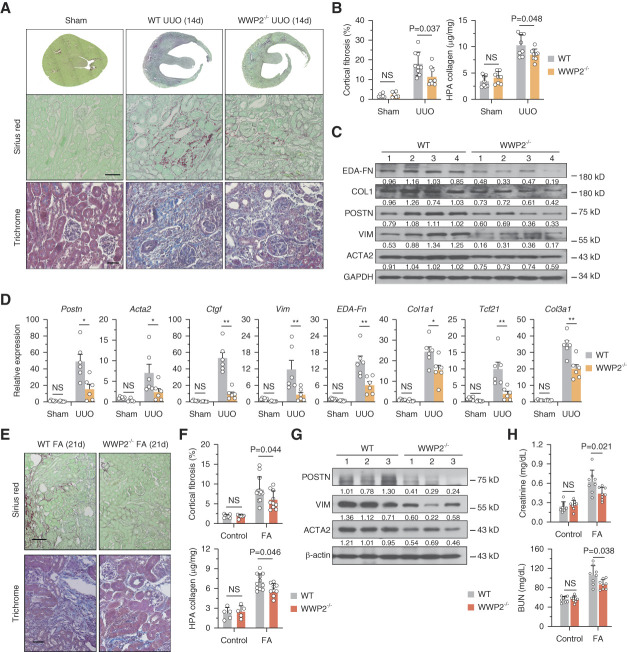
**WWP2 deficiency protects from renal fibrosis *in vivo*.** (A) Representative images of WT and WWP2^−/−^ mouse kidneys following UUO model for 14 days (*n*=8 images recorded for each condition). Top and middle panels: Sirius red staining for whole section and representative fibrotic area. Scale bars, 50 *μ*m. Bottom panels: representative images of Masson's trichrome staining for representative fibrotic area. Scale bars, 20 *μ*m. (B) Quantitative analysis of cortical fibrosis–positive area (left, %) and HPA collagen levels (right, *μ*g/mg) WT and WWP2^−/−^ mouse kidneys following UUO model for 14 days (*n*=6–10, in each experimental group). (C) Representative Western blot for ECM proteins in UUO kidney tissue from WT and WWP2^−/−^ mouse kidneys following UUO model for 14 days. (D) Expression of ECM genes, determined by RT-qPCR, in kidney from WT and WWP2^−/−^ mouse following UUO model for 14 days (*n*=6, in each group). (E) Representative images of folic acid–induced fibrotic kidneys at day 21 in WT and WWP2^−/−^ mice (*n*=6 images recorded for each condition). Top panel: Sirius red staining for representative fibrotic area. Scale bars, 50 *μ*m. Bottom panel: representative images of Masson's trichrome staining for representative fibrotic area. Scale bars, 20 *μ*m. The folic acid-induced fibrotic kidney model is abbreviated as FA. (F) Quantitative analysis of cortical fibrosis-positive area (%) and HPA collagen levels (*μ*g/mg) in folic acid–induced fibrotic kidneys in WT and WWP2^−/−^ mice (*n*=5–12, in each experimental group). (G) Representative Western blot for ECM proteins in folic acid–induced fibrotic kidneys from WT and WWP2^−/−^ mice. (H) Kidney function in the folic acid model as assessed by the level of plasma creatinine (mg/dl) and BUN (mg/dl) in both WT and WWP2^−/−^ mice (*n*=8 per experimental group). In each case, data values are reported as mean±SD, and *P* values were calculated by the two-tailed Mann–Whitney *U* test. NS, *P* > 0.1; *P < 0.05; **P < 0.01. ECM, extracellular matrix; FA, fatty acid; HPA, hydroxyproline assay; NS, not significant; WT, wild type.

### Quantification and Statistical Analysis

Data are expressed as mean±SD, unless otherwise indicated. The applied statistical tests (two-tailed nonparametric Mann–Whitney *U* test, two-tailed nonparametric Wilcoxon rank-sum test, the chi-squared test [for proportions]), which were dependent on the number of groups being compared and the study design, are indicated in each figure legend. R (v4.2.3) statistical programming language or SPSS (v26) Statistical Software Platform were used in all statistical analyses. All experiments requiring the use of animals, directly or as a source of cells, were subjected to randomization. The experimenters were blinded to the grouping information. All *in vivo* model analyses were conducted with sample sizes of at least *n*=8 independent mice. *In vitro* experiments were independently replicated a minimum of three times, as stated in the figure legends. Unless specified otherwise, each data point in the figures corresponds to an independent experiment, denoting either an individual mouse or cells isolated from individual mouse. For the Seahorse experiments, the data represent the readout from 3 to 4 microplate wells (technical replicates) and three different mice (biological replicates). Statistical significance is indicated by *P* value, where * denotes *P* < 0.05 and ** denotes *P* < 0.01 unless otherwise indicated. Multiple testing corrections (false discovery rate, BH-adjusted *P* value, Bonferroni-corrected *P* values) were implemented as required, as indicated in each case.

Raw (FASTQ files), preprocessed data (count matrix) and metadata of the scRNA-seq datasets, bulk RNA-seq data generated in primary myofibroblasts, and raw and processed bulk ChIP-seq data have been deposited in NCBI's Gene Expression Omnibus database (GSE241504). Scripts for the analysis pipeline used for single-cell clustering, data visualization, and generation of all major figures in this study were written in R, and code is available at Github (https://github.com/JingG/wwp2_kidney/).

## Results

### Association of Tubulointerstitial Expression of WWP2 and the Severity of Renal Fibrosis

In kidney biopsies from patients with CKD, WWP2 expression was more pronounced in kidneys with severe fibrosis compared with those with mild fibrosis (Figure 1A). Immunofluorescence staining showed that WWP2 was mainly expressed in tubular epithelial cells (Figure 1B, upper panels) as well as in interstitial myofibroblasts and colocalized with *α*-SMA-positive fibrotic kidney lesions (Figure 1B, lower panels). A significant correlation between the WWP2-positive area and fibrosis in the tubulointerstitial region was detected in a multicenter cohort of patients with CKD (*r*=0.274, *P* = 0.001, Figure 1C) and confirmed in two cohorts of patients with IgA nephropathy (Supplemental Figure 1B). We also detected a positive correlation between WWP2 expression in the tubulointerstitial region and the impairment of kidney function, that is, BUN and serum creatinine levels^59^ (Supplemental Figure 1C). Patients with moderate CKD (stage 3–4) showed higher expression of interstitial WWP2 when compared with those having mild CKD (stage 1–2), regardless of the original insult leading to the kidney damage (Figure 1D). There was no significant association between the glomerular WWP2 expression and the extent of glomerular fibrosis,^60^ kidney function, and proteinuria (Supplemental Figure 1, E–H).

**Figure 3:**
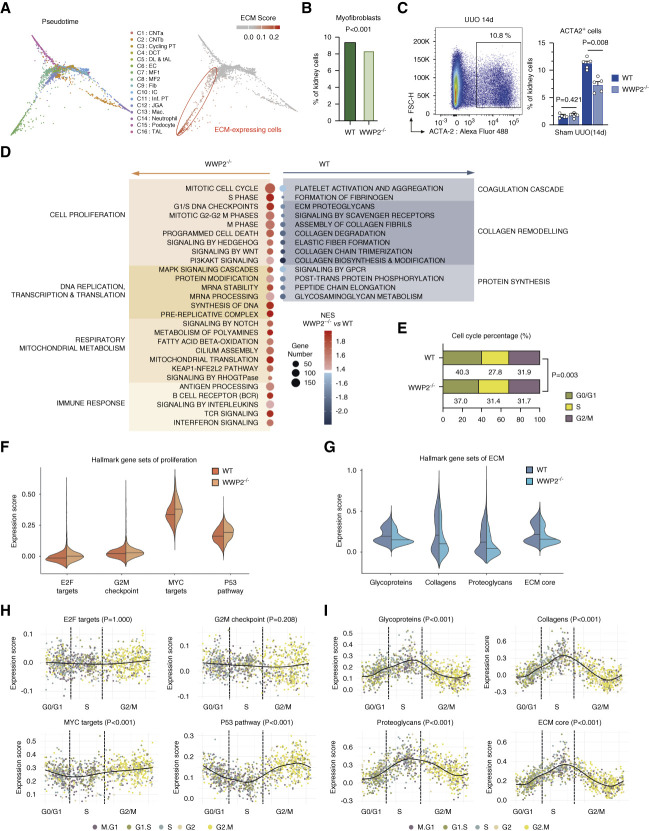
**WWP2 mediates myofibroblasts phenotypes in fibrotic kidneys.** (A) Diffusion map embedding of single cells data from UUO kidneys (left), and relative expression of ECM genes over imposed on the same embedding (right). Compared with other cell types, two myofibroblasts clusters (C7 and C8) presented high expression scores of ECM genes, quantified by a composite “ECM score” (see Methods). (B) Bar plots showing the percentage of myofibroblasts derived from UUO kidneys in WT and WWP2^−/−^ mice. Myofibroblasts percentage was calculated with respect to all renal cells, and *P* value was calculated by the chi-squared test (df=1) for cell proportions. (C) Left: representative graph of ACTA2^+^ cells in WT mouse using flow cytometry in renal living cells from UUO kidney (14 days). Right: quantification of ACTA2^+^ cells in kidney cells from WT and WWP2^−/−^ mice (*n*=5, from three independent experiments). Values are reported as mean±SD, and *P* values were calculated by the two-tailed Mann–Whitney *U* test. (D) Classification of reactome pathways enriched in ECM-expressing myofibroblasts and significantly different between WT and WWP2^−/−^ UUO kidneys by GSEA (FDR <0.05). NES, where a positive NES indicates upregulation in WWP2^−/−^ compared with WT myofibroblasts. (E) Proportions of ECM-expressing myofibroblasts at different phases of the cell cycle, grouped as G0/G1, S, and G2/M phases. ECM-expressing myofibroblasts from UUO kidneys are grouped according to WT and WWP2^−/−^ genotypes. *P* value was calculated by the chi-squared test for cell number in G0/G1, S, and G2/M phases, yielding χ^2^=11.89, df=2, *P* value = 0.003. (F and G) Expression score for hallmark gene sets for proliferation (F) and ECM production genes (G) in ECM-expressing myofibroblasts from UUO fibrotic kidneys. See Methods for definition of gene sets and score calculation. For each gene set, difference in expression score between WT and WWP2^−/−^ groups was tested using the nonparametric Wilcoxon rank-sum test; for each given gene set, the *P* value for the difference was < 0.001. (H and I) Using the Revelio algorithm (see Methods), myofibroblast cells derived from five human CKD kidneys^10^ are arranged along a pseudotime trajectory based on to their cell cycle phases and grouped as G0/G1, S, and G2/M. The distribution of hallmark gene set scores for proliferation (H) and ECM production (I) is shown for each myofibroblast arranged accordingly to its cell cycle phase, and the main trend is approximated by smooth line interpolation (bold black line). For each gene set, *P* values for significance of change in the linear trend (bold black line) were calculated by local regression-based WAVK test (see Methods for additional details). FDR, false discovery rate; GSEA, gene set enrichment analysis, NES, normalized enrichment score.

In the UUO model, a commonly used preclinical murine model for tubulointerstitial fibrosis,^61^ the intensity of WWP2 staining was higher in fibrotic kidneys compared with control ones (Figure 1E), which was localized to tubular cells (Figure 1F, left panels) and interstitial myofibroblasts (Figure 1F, right panels). This was supported by the relatively increased protein and transcript expression levels of WWP2 in fibrotic kidneys (Figure 1G). We generated a transgenic mouse line engineered to overexpress WWP2 (WWP2^Tg^) (Supplemental Figure 2A). Compared with controls (Control^Tg^), WWP2^Tg^ mice exhibited higher percentage of fibrosis in the kidney sections (Supplemental Figure 2B). WWP2 overexpression also increased fibrotic ECM marker genes in UUO kidneys, as shown by the upregulation of ACTA2, COL3, and DEA-FN (Supplemental Figure 2C).

### WWP2 Deficiency Protected from Renal Fibrosis *In Vivo*

To investigate the effect of WWP2 deficiency on renal fibrosis, we used a previously generated WWP2^−/−^ mouse strain^28^ and confirmed the deletion of WWP2-FL and -N isoforms in kidneys (Supplemental Figure 3A). After 14 days of UUO, there was significantly mitigated renal fibrosis in WWP2^−/−^ mice (Figure 2A), which showed approximately 50% reduction in cortical fibrosis and decreased collagen deposition compared with UUO WT mice (Figure 2B). In line with the biological analysis, WWP2 deficiency attenuated the upregulation of ECM marker genes in UUO-induced kidneys at both protein and mRNA levels (Figure 2, C and D). However, the effect of WWP2 deficiency on interstitial fibrosis was not apparent at 7 days of UUO, a time point that coincides with mild–moderate renal fibrosis (Supplemental Figure 3, B–E).

**Figure 4:**
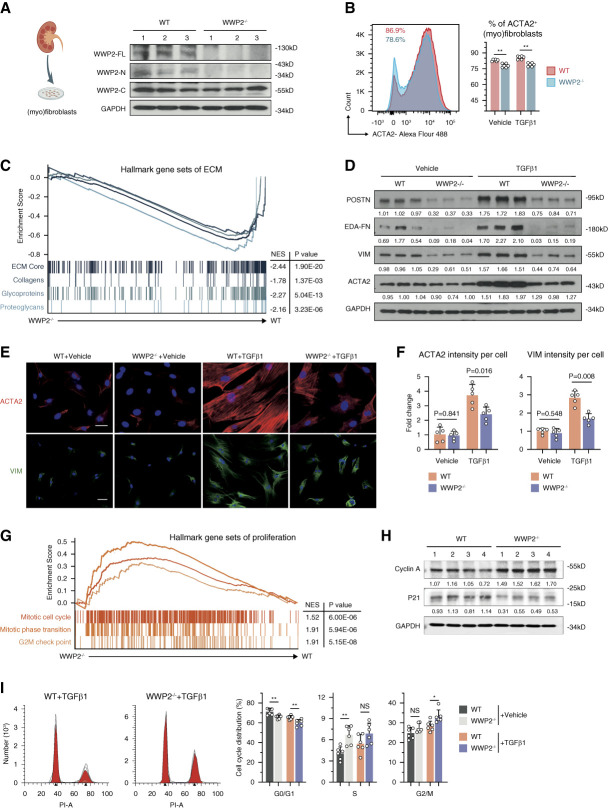
**WWP2 deficiency promotes cellular proliferation and supresses profibrotic activation in renal myofibroblasts *in vitro*.** (A) Representative Western blot showing the levels of WWP2 in primary cultured renal myofibroblasts (P2) derived from WT and WWP2^−/−^ kidneys. WWP2^−/−^ cells lack WWP2 full-length and -N isoforms expression. (B) Left: representative graph of ACTA2^+^ cells in cultured renal myofibroblasts (P2) derived from WT and WWP2^−/−^ mice using flow cytometry. Right: quantification of ACTA2+ cells in cultured myofibroblasts derived from WT and WWP2^−/−^ kidneys (*n*=6, from three independent experiments). Values are reported as mean±SD. TGF*β*1 (5 ng/*μ*l) for 72 hours. (C) GSEA of ECM pathways in cultured renal myofibroblasts, treated with TGF*β*1 (5 ng/*μ*l) for 72 hours, showing the enrichment score for ECM gene sets in WWP2^−/−^ (*x* axis, left) and WT kidneys (*x* axis, right). NES, where negative values indicate downregulation of the gene set in WWP2^−/−^ myofibroblasts with respect to WT myofibroblasts. (D) Representative Western blotting for ECM proteins in cultured TGF*β*1-treated myofibroblasts (P2) derived from WT and WWP2^−/−^ kidneys. TGF*β*1 (5 ng/*μ*l) for 72 hours. (E and F) Representative microscopy images (E) and quantification analysis (F) with immunostaining for ACTA2 and VIM in cultured renal TGF*β*1-treated myofibroblasts derived from WT and WWP2^−/−^ kidneys (*n*=5 independent experiments). One single dot indicates the average of 25–40 myofibroblasts taken from each slide. Values are reported as mean±SD. TGF*β*1 (5 ng/*μ*l) for 72 hours. (G) Similar to the data in (C), GSEA of proliferation pathways in cultured renal myofibroblasts (P2) treated with TGF*β*1 (5 ng/*μ*l) for 72 hours. Enrichment score comparing WWP2^−/−^ and WT myofibroblasts. Positive NES values indicate upregulation in WWP2^−/−^ myofibroblasts with respect to WT myofibroblasts. (H) Representative Western blotting for cyclin A and P21 in cultured TGF*β*1-treated renal myofibroblasts (P2) derived from WT and WWP2^−/−^ kidneys. TGF*β*1 (5 ng/*μ*l) for 24 hours. (I) Left: representative graph of cell cycle in cultured TGF*β*1-treated renal myofibroblasts (P2) derived from WT and WWP2^−/−^ mice using flow cytometry. Right: quantification of cell cycle at G0/G1, S, and G2/M phases in cultured myofibroblasts derived from WT and WWP2^−/−^ kidneys (*n*=6, from three independent experiments). TGF*β*1 (5 ng/*μ*l) for 24 hours. Values are reported as mean±SD. *P < 0.05; **P < 0.01.

The protective effect of WWP2 deficiency was confirmed in the folic acid–induced nephropathy model.^62^ Twenty-one days after folic acid induction, histochemistry for Sirius red and Masson's trichrome showed reduced fibrotic lesions, associated with a lower percentage of cortical fibrosis and collagen content in WWP2^−/−^ when compared with WT controls (Figure 2, E and F). The expression of ECM proteins in the kidney was also reduced in WWP2^−/−^ mice (Figure 2G). Moreover, WWP2 deficiency protected from kidney dysfunction, as indicted by the lower levels of BUN and creatinine in plasma from WWP2^−/−^ mice (Figure 2H).

These data from two *in vivo* fibrotic models demonstrate the protective antifibrotic effect of WWP2 deficiency. Additional data in WWP2^Tg^ mice (Supplemental Figure 2) corroborate the role of WWP2 in renal fibrosis.

### WWP2 Uncoupled Myofibroblast Activation from Proliferation in the Fibrotic Kidney

We previously demonstrated that WWP2 regulates heart fibrosis and cardiac fibroblast activation.^28^ Since fibroblast activation is coupled to their proliferative status, we next investigated whether the stromal cell heterogeneity reflect different states of cell activation/proliferation. We thus performed single-cell transcriptional analysis, which identified two cell populations (clusters 7–8) functionally enriched for ECM organization (Supplemental Figure 4, A and B). Pseudotime analysis of the trajectory of gene expression changes confirmed that clusters 7–8 were the main source of ECM gene expression in UUO fibrotic kidney (Figure 3A). Since clusters 7–8 were enriched for markers of myofibroblasts, and around 40% of these cells expressed Acta2 (Supplemental Figure 4C), we defined these clusters as renal myofibroblasts. WWP2 regulated the proportion of myofibroblasts in UUO-induced fibrotic kidneys, that is, a lower percentage of clusters 7–8 in WWP2^−/−^ mice compared with WT mice (Figure 3B), which was supported by flow cytometry analysis that showed a significantly reduced proportion of ACTA2^+^ cells (Figure 3C). Comparatively, cluster 7 is enriched for processes related to collagen synthesis, while cluster 8 is enriched for genes related to cell proliferation and TCA cycle (Supplemental Figure 4D). Compared with WT cells, WWP2^−/−^ myofibroblasts contain relatively less cluster 7 and more cluster 8 cells (Supplemental Figure 4E).

We further used GSEA to map the functional pathways modulated by WWP2 (Figure 3D). Renal myofibroblasts from WWP2^−/−^ UUO mice showed increased expression of genes related to mitotic cell cycle, cell division phases, DNA replication, transcription, and translation, which were previously reported to contribute to cellular proliferation.^63^ In keeping with this, WWP2^−/−^ myofibroblasts had a lower proportion of cells in G0/G1 cell cycle phases compared with WT mice in fibrotic kidneys (Figure 3E), and higher expression of hallmark gene sets for cell proliferation (*P* < 0.001 for each gene set, Figure 3F). By contrast, the expression of ECM pathways, particularly collagen production/remodeling, was significantly reduced in WWP2^−/−^ myofibroblasts (Figure 3D). The gene sets related to core ECM matrisome were also downregulated in WWP2^−/−^ myofibroblasts compared with WT cells (*P* < 0.001 for each gene set, Figure 3G).

In myofibroblasts from human CKD kidneys, cellular proliferation and profibrotic activation were also differentially active across cell cycle phases. Using the Revelio algorithm (see Methods), we extracted cell cycle information for renal myofibroblasts derived from ten patients with CKD^10^ and focused on cell proliferation and ECM processes. The proliferating MYC targets and P53 pathway gene sets exhibited cell cycle–dependent transcriptional changes, with the highest expression observed at G2/M phase (Figure 3H), while all ECM gene sets showed the lowest expression at G2/M phase (Figure 3I).

Thus, single-cell analysis demonstrated heterogeneity of renal myofibroblasts populations in fibrotic kidney, indicating cell proliferation and profibrotic activation, respectively. Both are regulated by WWP2 deficiency, which promotes cellular proliferation while inhibiting collagen synthesis. In addition, considering the expression level of WWP2 in tubular cells (Figure 1, B and E), we explored the potential profibrotic effect of tubular cells signaling on myofibroblasts (Supplemental Figure 5). *WWP2* has similar expression in myofibroblasts and tubular cells, but less genes are regulated by WWP2 in tubular cells (*i.e*., less differentially expressed genes between WT and WWP2^−/−^) (Supplemental Figure 5, A and B). Functionally, WWP2 deficiency upregulated pathways mainly related to cell homeostasis, but not inflammation, and moderately suppressed pathways related the ECM organization (Supplemental Figure 5C). Moreover, tubular cells and myofibroblasts crosstalk was not affected by WWP2 deficiency, as shown by the similar activity of ligand–receptor interactions in WWP2^−/−^ and WT, as well as the similar mRNA expression of ligands in tubular cells (Supplemental Figure 5, D and E).

### WWP2 Promoted Fibrotic Activation and Suppresses Proliferation of Myofibroblasts *In Vitro*

The regulation of myofibroblasts by WWP2 was further characterized by culturing primary renal fibroblasts isolated from WT and WWP2^−/−^ mice. WWP2^−/−^ cells lack WWP2 expression (Figure 4A), and TGF*β*1 treatment upregulates WWP2 during myofibroblasts differentiation (Supplemental Figure 6A). Flow cytometry analysis showed that the majority of cultured WT myofibroblasts express ACTA2, and the fraction of ACTA2-expressing cells was reduced in WWP2^−/−^ myofibroblasts (Figure 4B).

**Figure 5:**
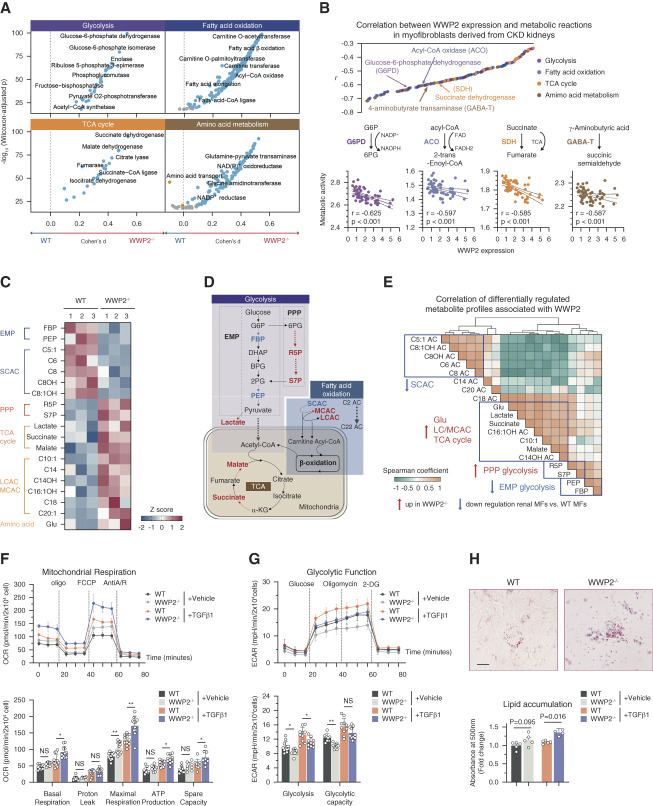
**Differential energy metabolism of WT and WWP2**^**−/−**^ **myofibroblasts.** (A) Compass score differential activity test in ECM-expressing myofibroblasts derived from WT and WWP2^−/−^ UUO kidneys, for reactions in the glycolysis, TCA cycle, fatty acid oxidation, and amino acid metabolism pathways. Statistical significance (*y* axis) for the difference in the activities scores of metabolic resections between WWP2^−/−^ and WT group was assessed by the nonparametric Wilcoxon rank-sum test, while the effect size was estimated by Cohen's D (*x* axis). The whole set of metabolic reactions changes in myofibroblasts are in Supplemental Figure 6A. (B) WWP2 mRNA expression is negatively associated with activity of metabolic resections in renal myofibroblasts from five human CKD patients' kidneys.^10^ Upper panel: significant Spearman correlations (*P* < 0.05) between compass scores of metabolic resections and the expression level of WWP2 in renal myofibroblasts. The color coding represents different metabolic pathways of the metabolic reactions. Lower panels: detail on the correlation between WWP2 expression and activity of four key metabolic reactions. The data for whole set of metabolic reactions is showed in Supplemental Figure 6B. (C) Metabolomics analysis shows the profile of metabolites exhibiting significant differences (*P* < 0.05, FDR <17%, two-tailed nonparametric Mann–Whitney *U* test) between cultured TGF*β*1-treated (5 ng/*μ*l for 72 hours) renal myofibroblasts (P2) derived from WT and WWP2^−/−^ kidneys. Each metabolite level was normalized and represented as Z-score. (D) Simplified overview of central metabolic fluxes in glycolysis, TCA cycle, fatty acid oxidation, and amino acid metabolism in cultured renal myofibroblasts derived from WT and WWP2^−/−^ kidneys. Color indicates the relative metabolite or enzyme rates in WWP2^−/−^ myofibroblasts compared with WT cells, where red indicates higher flux in WWP2^−/−^ myofibroblasts and blue indicates higher flux in WT cells. (E) Coordinated regulation of metabolites associated with WWP2 deficiency. Metabolite–metabolite correlation analysis of differential metabolites (reported in C). Positive correlations are depicted in orange, while negative correlations are represented in green. Metabolites that exhibit a high degree of correlation are highlighted (blue rectangles). (F) Upper panel: representative Seahorse Mito stress assays for the OCR in cultured TGF*β*1-treated (5 ng/*μ*l for 72 hours) renal myofibroblasts derived from WT and WWP2^−/−^ kidneys. Lower panel: barplots summarizing the phenotypes derived from OCR analysis. *n*=3 independent experiments, each containing readouts from 3 to 4 microplate wells (technical replicates). (G) Upper panel: representative Seahorse Glycolysis assays for the ECAR in cultured renal myofibroblasts derived from WT and WWP2^−/−^ kidneys. Lower panel: barplots summarizing the phenotypes derived from ECAR analysis. *n*=3 independent experiments, each containing readouts from 3 to 4 microplate wells (technical replicates). (H) Visualization (upper panels) and quantification (lower panels) of neutral lipids by oil red O analysis in cultured TGF*β*1-treated (5 ng/*μ*l for 72 hours) renal myofibroblasts derived from WT and WWP2^−/−^ kidneys. Scale bars, 50 *μ*m. (*n*=5 independent experiments.) Data values are reported as mean±SD. *P* values were calculated by two-tailed Mann–Whitney *U* test. *P < 0.05; **P < 0.01. *α*-KG, *α*-ketoglutarate; 2PG, 2-phosphoglycerate; BPG, bisphosphoglycerate or 2,3-bisphosphoglycerate; DHAP, dihydroxyacetone phosphate; ECAR, extracellular acidification rate; EMP, Embden-Meyerhof-Parnas pathway of glycolysis; FBP, fructose-1,6-bisphosphate; G6P, glucose-6-phosphate; Glu, glutamic acid; LCAC, long chain of acylcarnitine; MCAC, medium chain of acylcarnitine; OCR, oxygen consumption rate; PEP, phosphoenolpyruvate; PPP, pentose phosphate pathway; R5P, ribose-5-phosphate; S7P, sedoheptulose-7-phosphate; SCAC, small chain of acylcarnitine.

In keeping with our scRNA-seq results in UUO kidneys (Figure 3G), bulk RNA sequencing analysis showed that cultured WWP2^−/−^ myofibroblasts exhibit a significantly decreased expression of ECM gene sets compared with WT myofibroblasts (Figure 4C). On TGF*β*1 treatment, the increased expression of fibrous proteins, such as ACTA2, extra domain A fibronectin and periostin, was largely reduced in WWP2^−/−^ myofibroblasts (Figure 4D), with a diffuse expression and rare incorporation into stress fibers (Figure 4, E and F). Moreover, cell proliferation gene sets were more prominently expressed in WWP2^−/−^ myofibroblasts compared with WT (Figure 4G), which also confirmed our previous results (Figure 3F). The expression level of cell division phase checkpoint proteins P21 and Cyclin A in cultured myofibroblasts was also regulated by WWP2 (Figure 4H). Cell cycle analysis showed that the majority of cultured renal myofibroblasts were in G0/G1 phase (approximately 70%), which was slightly decreased after TGF*β*1 treatment and further reduced by WWP2 deficiency, while cells in S or G2/M phase showed the opposite trend (Figure 4I). Consequently, WWP2^−/−^ renal myofibroblasts showed higher cell proliferation compared with WT cells, irrespective of TGF*β*1 treatment (Supplemental Figure 6B).

We generated WWP2 overexpressing myofibroblasts (WWP2^OE^) (Supplemental Figure 6C), which confirmed that WWP2 promoted fibrotic activation and suppressed myofibroblasts proliferation. WWP2^OE^ led to further enhanced production of fibronectin 1 in myofibroblasts on TGF*β*1 treatment (Supplemental Figure 6D) and lower percentage of myofibroblasts in G2/M phase (Supplemental Figure 6E).

### WWP2 Regulates Myofibroblast Energy Metabolism during Fibrosis

To further investigate a potential metabolic regulatory role of WWP2 (Figure 3D), we used compass^53^ to profile the metabolic reactions in profibrotic myofibroblasts (see Methods). In murine renal myofibroblasts, WWP2 deficiency yielded increased activity in a wide range of metabolic reactions (Figure 5A and Supplemental Figure 7A), especially those related to central carbon and amino acid metabolism. These data suggest that WWP2 deficiency is associated with higher glucose and lipid utilization, including pentose phosphate pathway and fatty acid oxidation. WWP2 also might affect amino acid metabolism, which contributes to the balance of the NAD(P)^+^/NAD(P)H. WWP2 deficiency might enhance TCA cycle activity and mitochondrial respiratory metabolism, which we corroborated by analysis of renal myofibroblasts from patients with CKD: *WWP2* expression was negatively associated with reactions necessary for mitochondrial energy metabolism (Figure 5B, top panel and Supplemental Figure 7B). Our analyses emphasize several reactions regulated by WWP2 (Figure 5B, bottom panels): glucose-6-phosphate dehydrogenase catalyzed reaction, the first step in the pentose phosphate pathway,^64^ which leads to the conversion of NADP to NADPH^65^; Acyl-CoA oxidase, responsible for fatty acid oxidation, acting on CoA derivatives of fatty acids with aliphatic carbons between medium to long chain^66^; 4-aminobutyrate transaminase tightly coupled to glutamate metabolism and cellular energy metabolism^67^; and succinate dehydrogenase as part of the TCA cycle.^68^ The association of *WWP2* expression in renal myofibroblasts and these metabolic reactions linked to mitochondrial respiration was confirmed in a second CKD cohort (Supplemental Figure 8, A and B). Such associations were stronger in patients with CKD compared with controls (Supplemental Figure 8C), suggesting that the downregulation of these metabolic reactions by WWP2 may be more pronounced during CKD.

**Figure 6:**
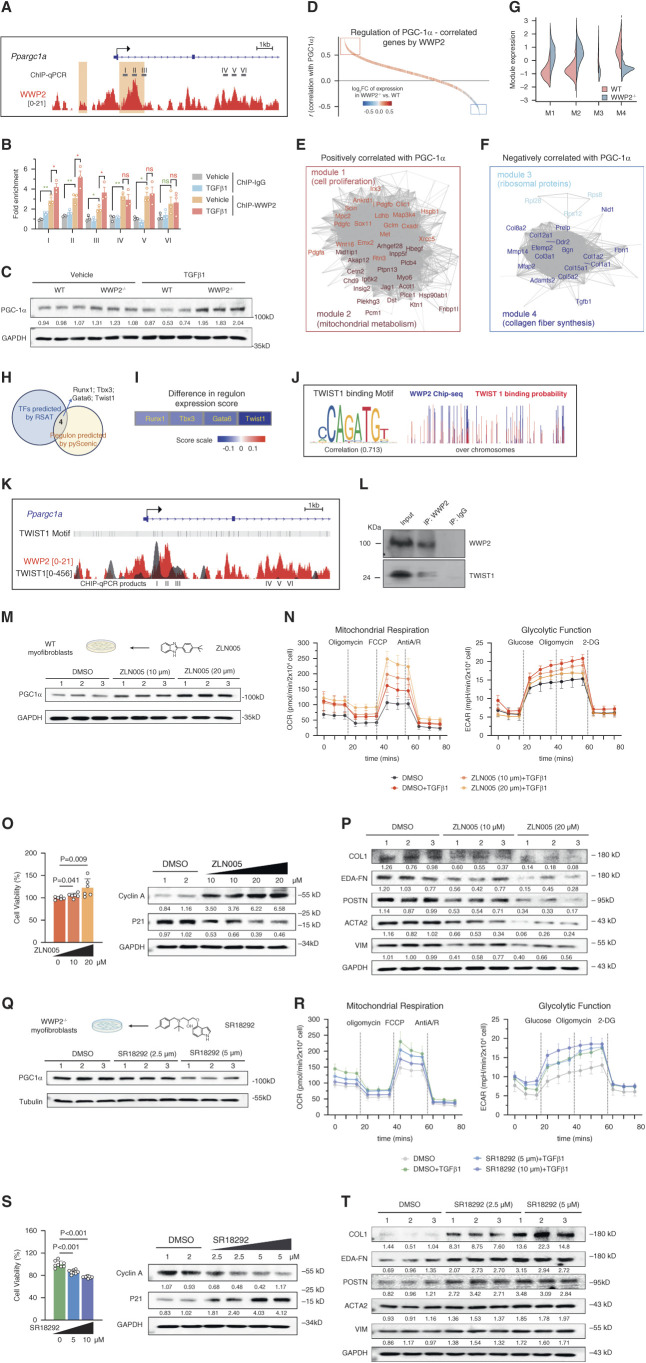
**WWP2 targets TWIST1 and regulates PGC-1*α*–mediated mitochondrial respiratory function.** (A) Distribution of WWP2 ChIP-seq signal (red) at the *Ppargc1a* locus in primary WT myofibroblasts after TGF*β*1 stimulation (5 ng/*μ*l, 72 hours). The primers designed for ChIP-qPCR (gray boxes, I–VI) and the reference *Ppargc1a* gene (blue) are indicated. Genomics regions I–III contain WWP2-binding signals (ChIP-Seq peaks, in red), which overlap with known DNA-binding elements (light yellow boxes) identified from the REMAP DB^56^; regions IV–VI serve as controls (*i.e*., no DNA-binding elements). (B) ChIP-qPCR analysis shows increased binding of WWP2 complex at *Ppargc1a* genomic regions I, II, and III after TGF*β*1 stimulation, which overlap with predicted DNA-binding elements. Each bar shows WWP2 enrichment normalized to input and the ChIP-IgG control. Values are reported as mean±SD (*n*=3, independent experiments). *P* values were calculated by two-tailed Mann–Whitney *U* test. *P < 0.05; **P < 0.01; ns, *P* > 0.05. (C) Representative Western blot for PGC-1*α* in cultured renal myofibroblasts derived from WT and WWP2^−/−^ kidneys. TGF*β*1 stimulation (5 ng/*μ*l) for 24 hours. (D) Genome-wide distribution of genes sorted based on their Pearson correlation with PGC-1*α* expression in myofibroblasts derived from fibrotic mouse kidneys. The color scale represents the fold change (log_2_FC) in expression between WWP2^−/−^ myofibroblasts compared with WT cells. The red rectangle includes genes positively related to PGC-1*α* (*r*>0.5, *P* < 0.001) and which are upregulated in WWP2^−/−^ myofibroblasts compared with WT cells; the genes in the blue rectangle are negatively related to PGC-1*α* (*r*<−0.5, *P* < 0.001) and downregulated in WWP2^−/−^ myofibroblasts. (E and F) Subset of network genes from those highlighted in the red (D) and blue (E) rectangles in (C), which are correlated with PGC-1*α* (|r|>0.6). PGC-1*α*-network genes are further clustered in four distinct modules (M1–M4), positively (M1–M2) and negatively (M3–M4) correlated with PGC-1*α*, respectively (see Methods for details). Within each network, edges represent positive coexpression relationships between genes, and colors indicate functional clustering of the module genes, highlighting the cell proliferation (M1) and mitochondrial metabolism (M2) (D), and processes related to fibrous protein synthesis (M3 and M4, E). The genes highlighted are more strongly correlated positively (*r*>0.8, E) or negatively (*r*<−0.8, F) with PGC-1*α* expression, respectively, in myofibroblasts for fibrotic mouse kidneys. (G) For each module (M1–M4, see E and F), violin plot shows the distribution of normalized gene expression levels derived from bulk-RNA seq analysis of cultured WT and WWP2^−/−^ myofibroblasts treated with TGF*β*1 (5 ng/*μ*l, 72 hours). For each module, *P* value for the difference between WT and WWP2^−/−^ myofibroblasts was < 0.001, which was calculated by the two-tailed Wilcoxon rank-sum test. (H) Venn diagram showing the overlapping transcriptional factors identified by CHIP-seq motif analysis by RSATs^57^ (Supplemental Table 2) and scRNA-seq regulon analysis by pySCENIC analyses^58^ (Supplemental Table 4). (I) The difference in expression score of each regulon between WWP2^−/−^ and WT myofibroblasts. The TWIST1 regulon showed the largest and most significant expression score difference between WWP2^−/−^ and WT myofibroblasts. (J) DNA-binding sequence motif of TWIST1 (left panel), and correlation between WWP2-binding signaling (blue) and TWIST-binding probability (red) (right panel). (K) Distribution of WWP2 ChIP-seq signal (red) at the Ppargc1a locus in primary WT myofibroblasts after TGF*β*1 stimulation (5 ng/*μ*l, 72 hours). The location of TWIST1-binding sequence motifs (gray vertical lines) and TWIST1-binding signals (gray) identified from the REMAP DB^56^ are also indicated. (L) Representative Western blot image of co-IP showing a direct interaction of WWP2 full-length isoform and TWIST1 in myofibroblasts. (M) Effects of an activator of PGC-1*α*, ZLN005 (10 and 20 *μ*M), in cultured WT myofibroblasts after TGF*β*1 treatment (5 ng/*μ*l, 72 hours). DMSO (10 *μ*M) was used as a reference control. Upper panel: experimental design schematic. Lower panel: representative Western blot showing the effect of ZLN005 on PGC-1*α* protein expression in cultured WT myofibroblasts. (N) Effects of ZLN005 (10 and 20 *μ*M) on cell metabolism in cultured WT myofibroblasts after TGF*β*1 treatment (5 ng/*μ*l, 72 hours), measured by Seahorse assay. DMSO (10 *μ*M) was used as a reference control. Left panel: experimental design schematic. Middle panel: representative Seahorse Mito stress assays for OCR. Right panel: representative Seahorse Glycolysis assays for ECAR. *n*=3 independent experiments, each containing readouts from 3 to 4 microplate wells (technical replicates). Results of quantification analysis are shown in Supplemental Figure 9C. (O) Effects of ZLN005 (10 and 20 *μ*M) on cell proliferation in cultured WT myofibroblasts after TGF*β*1 treatment (5 ng/*μ*l, 72 hours), measured by MTS assay (left panel) and Western blot (right panel) (*n*=6, from three independent experiments). *P* values were calculated by the two-tailed Mann–Whitney *U* test. (P) Western blot showing the effect of ZLN005 (10 and 20 *μ*M) on ECM proteins production in cultured WT myofibroblasts after TGF*β*1 treatment (5 ng/*μ*l, 72 hours). (Q) Effects of an inhibitor of PGC-1*α*, SR18292 (2.5 and 5 *μ*M), in cultured WWP2^−/−^ myofibroblasts after TGF*β*1 treatment (5 ng/*μ*l, 72 hours). DMSO (5 *μ*M) was used as a reference control. Upper panel: experimental design schematic. Lower panel: representative Western blot showing the effect of SR18292 on PGC-1*α* protein expression in cultured WWP2^−/−^ myofibroblasts. (R) Effects of SR18292 (2.5 and 5 *μ*M) on cell metabolism in cultured WWP2^−/−^ myofibroblasts after TGF*β*1 treatment (5 ng/*μ*l, 72 hours) by Seahorse assay. DMSO (5 *μ*M) was used as a reference control. Left panel: experimental design schematic. Middle panel: representative Seahorse Mito stress assays for OCR. Right panel: representative Seahorse Glycolysis assays for ECAR. *n*=3 independent experiments, each containing readouts from 3 to 4 microplate wells (technical replicates). Results of quantification analysis are shown in Supplemental Figure 9D. (S) Effects of SR18292 (2.5 and 5 *μ*M) on cell proliferation in cultured WWP2^−/−^ myofibroblasts after TGF*β*1 treatment (5 ng/*μ*l, 72 hours), measured by MTS assay (left panel) and Western blot (right panel) (*n*=6, from three independent experiments). *P* values were calculated by the two-tailed Mann–Whitney *U* test. (T) Western blot showing the effect of SR18292 (2.5 and 5 *μ*M) on ECM proteins production in cultured WWP2^−/−^ myofibroblasts after TGF*β*1 treatment (5 ng/*μ*l, 72 hours). 2-DG, 2-deoxy-D-glucose; ChIP, chromatin immuno-precipitation; ChIP-qPCR, chromatin immunoprecipitation quantitative PCR; ChIP-seq, chromatin immunoprecipitation sequencing; EDA-FN, extra domain A fibronectin; FCCP, fluoro-carbonyl cyanide phenylhydrazone; MTS, 3-(4,5-dimethylthiazol-2-yl)-5-(3-carboxymethoxyphenyl)-2-(4-sulfophenyl)-2H-tetrazolium; PGC-1α, peroxisome proliferator-activated receptor gamma coactivator 1-alpha; POSTN, periostin; RNA-seq, RNA sequencing; RSAT, regulatory sequence analysis tool; scRNA-seq, single-cell RNA sequencing.

We then took a metabolomics approach to detail the regulation of WWP2 on renal myofibroblasts energy metabolism (Figure 5, C and D, and Supplemental Figure 9). WWP2^−/−^ myofibroblasts showed increased ribose-5-phosphate and sedoheptulose-7-phosphate in the pentose phosphate pathway, at the expense of fructose 1,6-bisphosphate and phosphoenolpyruvate in the Embden-Meyerhof-Parnas pathway. WWP2^−/−^ myofibroblasts also showed changes in AC facilitating fatty acid oxidation,^69^ including an increase in long/medium chain AC and a decrease in small chain AC. The higher levels of lactate, malate, and succinate confirmed an enhanced TCA cycle in WWP2^−/−^ myofibroblasts. These metabolites were coregulated by WWP2 (Figure 5E), implying a synergistic effect of WWP2 deletion on multiple interconnected metabolic processes.

Given that TCA cycle is coupled with OXPHOS to produce ATP, we next sought to evaluate mitochondrial function by Seahorse analysis (Figure 5, F and G). Compared with WT cells, WWP2^−/−^ myofibroblasts showed a higher oxygen consumption rate, with increased levels of basic respiration, maximum respiration, and ATP production (Figure 5F), and exhibited a decreased ECAR (Figure 5G). These results confirmed the increased fatty acid oxidation and TCA cycle activity observed by metabolomics (Figure 5C). WWP2^−/−^ myofibroblasts also show significantly higher lipid droplet accumulation (Figure 5H), suggestive of higher fatty acid uptake, and possibly contributing to increased fatty acid oxidation. Taken together, these data show that WWP2 regulates myofibroblast energy metabolism (Supplemental Figure 9E).

### PGC-1*α* Signaling Mediates the WWP2 Regulated Myofibroblasts Metabolism

WWP2 has been reported to act as a cofactor in nuclear transcriptional complexes,^28,70^ and we showed its nuclear localization changes in myofibroblasts on TGF*β*1 treatment (Supplemental Figure 10A). We previously demonstrated that WWP2 regulates TF activity of SMAD2 in cardiac fibroblast^28^ and of IRF7 in cardiac macrophages.^29^ Therefore, we hypothesize a potential transcriptional regulatory role of WWP2 in fibrotic renal myofibroblasts. Given the regulatory role of WWP2 in fatty acid oxidation during murine and human renal fibrosis, we focused on PGC-1*α*, a critical modulator of fatty acid oxidation^25,26^ and of metabolism during tissue fibrosis.^71–73^ To investigate the transcriptional regulation of PGC-1*α* by WWP2, we first used ChIP-seq analysis and assessed WWP2 binding to the *Ppargc1a* locus, which encodes for PGC-1*α* (Figure 6A). Chromatin immunoprecipitation quantitative PCR analysis confirmed a significant increase in the binding of the WWP2 transcriptional complex to the *Ppargc1a* locus at regions I–III, compared with the control regions IV–VI (Figure 6B). WWP2 deficiency was associated with increased expression of PGC-1*α* in cultured myofibroblasts at protein and mRNA levels (Figure 6C and Supplemental Figure 10B). These data suggest that WWP2 may inhibit the transcription of PGC-1*α* in activated myofibroblasts.

We further interrogated the transcriptional activation of the wider PGC-1*α* regulatory network by WWP2 in myofibroblasts using scRNA-seq data from fibrotic kidney. We derived the sets of genes coexpressed with *Ppargc1a* in myofibroblasts and looked at the effect of WWP2 deficiency (Figure 6D). The genes positively correlated with *Ppargc1a* were upregulated in WWP2^−/−^ myofibroblasts (Figure 6D, red box) and enriched for cell proliferation (Figure 6E, module 1) and mitochondrial metabolism (Figure 6E, module 2). By contrast, genes negatively correlated with *Ppargc1a* were downregulated in WWP2^−/−^ myofibroblasts (Figure 6D, blue box); these were enriched for collagen synthesis, including ribosomal protein (Figure 6F, module 3) and collagen fiber genes (Figure 6F, module 4). Bulk RNA-seq analysis of TGF*β*1-stimulated renal myofibroblasts confirmed the upregulation of modules 1–2 and the downregulation of modules 3–4 in response to WWP2 deficiency (Figure 6G).

We further identified TWIST1 as a TF mediating the binding of the WWP2 transcriptional complex to *Ppargc1a* locus. RSAT analysis^57^ of our WWP2 ChIP-seq data predicted 14 distinct binding motif patterns encompassing 100 aligned IDs and identified 39 TFs or TF families as potential components of the transcriptional complex in conjunction with WWP2 (Supplemental Table 2). Based on scRNA-seq data, pySCENIC^58^ showed that WWP2 significantly regulates 35 TF networks (regulons) in myofibroblasts (Supplemental Table 4). Four transcriptional factors identified both by motif (RSAT) and regulon (pySCENIC) analysis were identified (Figure 6H), and in particular, the TWIST1 network was the most significantly regulated regulon (Figure 6I). The genome-wide distribution of TWIST1-binding motifs showed an overlap with the peak locations of the WWP2-binding complex (genome-wide correlation of TWIST1 and WWP2 binding r = 0.713, Figure 6J), including overlaps at the Ppargc1a locus (Figure 6K). Coimmunoprecipitation (Co-IP) confirmed the direct binding between WWP2 and TWIST1 in myofibroblasts under TGF*β*1 treatment (Figure 6L). These findings suggest that TWIST1 is involved in the WWP2-dependent regulation of PGC-1*α* transcription.

Moreover, in TGF*β*1-stimulated WT myofibroblasts, an activator of PGC-1*α*, ZLN005, upregulated PGC-1*α* expression in a dose-dependent manner (Figure 6M), leading to increased mitochondrial respiration and decreased glycolysis, as evidenced by elevated oxygen consumption rate levels and reduced ECAR levels, respectively (Figure 6N). ZLN005 treatment also enhanced myofibroblast cell proliferation (Figure 6O) and reduced profibrotic markers in myofibroblasts (Figure 6P). By contrast, an inhibitor of PGC-1*α*, SR18292, partially abrogates the increased metabolic response in myofibroblasts associated with WWP2 deficiency (Figure 6Q–R), which was mirrored by cell proliferation arrest and increased expression of profibrotic proteins (Figure 6T). The pharmacologic interventions targeting PGC-1*α* confirmed the contribution of WWP2-PGC-1*α*-axis in myofibroblasts (Supplemental Figure 10E).

All these results show that in profibrotic myofibroblasts, WWP2 modulates upstream the transcriptional activity of PGC-1*α*, a master regulator in mitochondria energy metabolism, particularly fatty acid oxidation^74^ and pentose phosphate pathway.^75^

## Discussion

Fibrogenesis is major driver of CKD progression, which is meditated by several renal cell types. Here, we took a myofibroblast-centric view on kidney tubulointerstitial fibrosis. We highlight the relevance of cell metabolism for myofibroblasts proliferation and profibrotic activation in CKD. In this context, we show that WWP2, an E3 ubiquitin ligase implicated in cardiac fibrosis,^28^ has an upstream regulatory role. Our work adds a previously unknown metabolic function of WWP2 in renal myofibroblasts and proposes WWP2 as a new druggable target to ameliorate renal fibrosis.^76^

Myofibroblasts are the main source of ECM and are crucial to the development of tissue fibrosis.^3^ During renal fibrosis, both cell proliferation and profibrotic activation^77^ contribute to the net ECM production in the tubulointerstitium and subsequent CKD progression.^17,23,78,79^ Activated profibrotic myofibroblasts are responsible for synthesizing fibrous proteins—the primary source of ECM production in fibrotic kidneys.^80^ Myofibroblasts have the capacity to proliferate in response to cytokine cues, leading to expansion of the fibroblast population in renal interstitial space.^12,81^ Here, we used scRNA-seq to identify functionally specialized myofibroblast populations in the fibrotic kidney and demonstrate their regulation by WWP2. We propose a model whereby profibrotic activation and proliferation of myofibroblasts are two interlinked states of cell polarization with differential outcome on ECM production—low cell proliferation correlating with high ECM production.^12,13^ WWP2 uncouples these two processes: WWP2 deficiency suppresses the profibrotic activation and enhances cell proliferation in both fibrotic kidneys and cultured myofibroblasts. Lemons *et al.*^19^ also reported similar observations with quiescent myofibroblasts exhibiting higher expression of fibrous proteins. We consolidate this observation and further reveal that renal myofibroblasts comprised a mixture of distinct subpopulations with different effector functions. In the fibrotic kidney, our single-cell analysis suggests the presence of at least two functionally distinct myofibroblasts subtypes (Supplemental Figure 4), and this inherent heterogeneity might explain the observed uncoupling of profibrotic activation and proliferation.^19,28,82^

Myofibroblast activation is typically driven by a variety of cytokines and growth factors produced by tubular cells, including TGF*β*1, Wnt ligands, platelet-derived growth factor, hepatocyte growth factor, fibroblast growth factors, and angiotensin.^83–87^ Moreover, WWP2 expression is also detected in the tubular cells of fibrotic kidneys (Figure 1), and its effect on these cells cannot be excluded. Despite the importance of tubular cells in renal fibrosis, our data suggest that the contribution of WWP2 in regulating these cells in fibrosis is limited, and this does not seem to occur through tubular proinflammatory activity or the secretion of profibrotic factors (Supplemental Figure 5). However, the mechanisms linking cell proliferation to profibrotic activation and tissue fibrosis remain to be elucidated using a myofibroblast-specific WWP2 knockout model.

During fibrotic activation and cell proliferation, myofibroblasts exhibit high metabolic activity.^19^ WWP2 regulates key metabolic processes in myofibroblasts, particularly those involved in mitochondrial respiratory function. In two CKD patient cohorts, *WWP2* expression is negatively associated with the activity of key metabolic reactions in myofibroblasts. This was confirmed in mice, as myofibroblasts derived from WWP2^−/−^ kidneys exhibited enhanced metabolic rates in central carbon metabolism and amino acid pathways. Important metabolic fluxes were increased in WWP2-deficient myofibroblasts, and these directly contribute to TCA cycle activity, ATP production, and mitochondrial function. In keeping with our findings, renal fibrosis is characterized by a decrease in mitochondrial biogenesis^88^ and by damage to mitochondrial DNA.^89^ Furthermore, the antifibrotic WWP2 deficiency resulted in a reduction in cell glycolysis, particularly *via* the Embden-Meyerhof-Parnas pathway. The regulation of this pathway by WWP2 is important, as inhibition of glycolysis has been reported to decrease renal fibrosis in UUO *via* proximal tubular cells–fibroblast crosstalk,^90^ as well as in myofibroblasts.^23^ Moreover, we showed how WWP2 deficiency upregulates the pentose phosphate pathway, including glucose-6-phosphate dehydrogenase–catalyzed reaction. This is the first step in the pentose phosphate pathway,^64^ which leads to the conversion of NADP^+^ to NADPH.^65^ The link between increased NADPH production and cell proliferation has been also reported in pulmonary myofibroblasts^20^ and cancer cells.^91^ In addition, although we did show major metabolic fluxes that are affected by WWP2 (*e.g*., fatty acid oxidation), other pathways and primary targets of WWP2 that lead to myofibroblast metabolic rewiring remain to be identified. A combination of lineage-tracing studies and cell type–specific WWP2 targeting can conclusively establish the broad significance of WWP2-mediated myofibroblast metabolism in renal fibrosis.

WWP2 can act as a co-TF,^28,70,92^ and here, we identify PGC-1*α* as a novel target of WWP2 in profibrotic myofibroblasts. Our analyses suggested TWIST1 as a potential TF interacting with WWP2 at the *Ppargc1a* locus. TWIST1 is a helix-loop-helix–containing TF involved in early development, apoptosis, cancer, and osteoblast differentiation.^93–95^ TWIST1 is also reported to downregulate the expression of PGC-1*α* in epithelial cells^96^ and skeletal muscle cells.^97^ Therefore, we propose that WWP2 is recruited to a transcriptional complex involving TWIST1 and that the complex binds to the regulatory locus of *Ppargc1a*.

PGC-1*α* is crucial for mitochondrial biogenesis and function,^98,99^ mitigating fibrosis progression.^100^ It also promotes tissue remodeling to a state that is metabolically more oxidative and less glycolytic.^101^ The regulation of myofibroblast metabolism by PGC-1*α* has been shown in wound healing, lung fibrosis, and cardiac remodeling, where it affects mitochondrial biogenesis, OXPHOS, and secretory state of myofibroblasts.^71–73^ Previous studies showed that PGC-1*α* contributes to fibrotic progression in multiple kidney disease models *via* its action outside the stromal compartment.^102–104^ Here, we show that PGC-1*α* in myofibroblasts stimulates the mitochondrial respiratory function and promotes cell proliferation, which is upregulated upstream by WWP2 deficiency. PGC-1*α* levels also exhibit a negative correlation with collagen synthesis, which was also suppressed by WWP2 deficiency. Using previously tested small molecules modulating PGC-1*α* activity, we showed that PGC-1*α* mediates, at least in part, the observed effects of WWP2 on myofibroblasts. While WWP2^92^ and PGC-1*α*^105^ have been both identified as Sox9-associated proteins during chondrogenesis, here, we show a negative regulatory relationship between WWP2 and PGC-1*α* in profibrotic myofibroblasts.

We demonstrate WWP2 as a potential druggable target for fibrosis progression in CKD. Inhibiting WWP2 might represent a promising therapeutic strategy for attenuating fibrosis progression. Given the paucity of actionable targets, our results might pave the way to design new therapeutic approaches in CKD.

## Supplementary Material

## Data Availability

Previously published data used for this study have been referenced with their original sources. Original data created for the study are available GSE241504 https://www.ncbi.nlm.nih.gov/geo/query/acc.cgi?acc=GSE241504.

## Supplemental Material

Supplemental Figure 1. Expression of WWP2 in the kidney of patients with CKD and UUO mice (related to Figure 1).

Supplemental Figure 2. WWP2 overexpression exacerbates renal fibrosis *in vivo* (related to Figure 1).

Supplemental Figure 3. Effect of WWP2 deficiency on renal fibrosis at 7 days of UUO (related to Figure 2).

Supplemental Figure 4. Single-cell transcriptomics landscape of kidney cells derived from UUO mice (related to Figure 3).

Supplemental Figure 5. Regulatory function of WWP2 in tubular cells derived from fibrotic kidneys.

Supplemental Figure 6. WWP2 overexpression mediates cellular proliferation and activation in renal myofibroblasts *in vitro* (related to Figure 4).

Supplemental Figure 7. Compass-based exploration of metabolic profile (related to Figure 5).

Supplemental Figure 8. Compass-based exploration of metabolic reactions regulated by WWP2 in another CKD cohort (related to Figure 5).

Supplemental Figure 9. Profile of metabolites measured in cultured renal myofibroblasts derived from WT and WWP2^−/−^ kidneys (related to Figure 5).

Supplemental Figure 10. Regulation of PGC-1*α* signaling by WWP2 (related to Figure 6).

Supplemental Dataset 1. Key resources used.

Supplemental Dataset 2. Uncut Western blots.

Supplemental Table 1. Details on the Bari CKD cohort.

Supplemental Table 2. TFBS motifs identified by RSAT using ENCODE and JASPAR database.

Supplemental Table 3. List of pathways tested significant (false discovery rate <0.05) by GSEA (Gene Set Enrichment Analysis) based on comparison of WWP2^−/−^ KO versus WT.

Supplemental Table 4. The transcription factor networks (regulons) regulated by WWP2 in myofibroblasts derived in fibrotic kidneys (upregulated and downregulated referring in WWP2^−/−^ versus WT, Benjamini–Hochberg adjust *P* < 0.05).
