# Supplementary material for: WWP2 Regulates Renal Fibrosis and the Metabolic Reprogramming of Profibrotic Myofibroblasts
Source: J Am Soc Nephrol. 2024 Mar 19;35(6):696–718. doi: 10.1681/ASN.0000000000000328 (PMC11164121; doi:10.1681/ASN.0000000000000328)

Supplemental Material Table of Contents:

Supplemental Figure 1. Expression of WWP2 in the kidney of patients with chronic kidney disease (CKD) and UUO-mice (related to Figure 1)

Supplemental Figure 2. WWP2 overexpression exacerbates renal fibrosis in vivo (related to Figure 1)

Supplemental Figure 3. Effect of WWP2 deficiency on renal fibrosis at 7 days of UUO (related to Figure 2)

Supplemental Figure 4. Single-cell transcriptomics landscape of kidney cells derived from UUO-mice (related to Figure 3)

Supplemental Figure 5. Regulatory function of WWP2 in tubular cells derived from fibrotic kidneys

Supplemental Figure 6. WWP2 overexpression mediates cellular proliferation and activation in renal myofibroblasts in vitro (related to Figure 4)

Supplemental Figure 7. Compass-based exploration of metabolic profile (related to Figure 5)

Supplemental Figure 8. Compass-based exploration of metabolic reactions regulated by WWP2 in another CKD cohort (related to Figure 5)

Supplemental Figure 9. Profile of metabolites measured in cultured renal myofibroblasts derived from WT and $WWP2^{-/-}$ kidneys (related to Figure 5)

Supplemental Figure 10. Regulation of PGC-1α signalling by WWP2 (related to Figure 6)

Supplemental Table 1. Details of two CKD cohorts

Supplemental Table 2. TFBS motifs identified by RSAT using JASPAR database

Supplemental Table 3. List of significant pathways (false discovery rate < 0.05) by GSEA (Gene Set Enrichment Analysis) based on comparison of $WWP2^{-/-}$ KO vs WT

Supplemental Table 4. The transcription factor networks (regulons) regulated by WWP2 in myofibroblasts derived in fibrotic kidneys

Supplemental Dataset 1. Uncut Western Blots

Supplemental Table 1. Details on the Bari CKD cohort

Supplemental Table 2. TFBS motifs identified by RSAT using ENCODE and JASPAR database

Supplemental Table 3. List of pathways tested significant (false discovery rate < 0.05) by GSEA (Gene Set Enrichment Analysis) based on comparison of WWP2-/- KO vesus WT

Supplemental Table 4. The transcription factor networks (regulons) regulated by WWP2 in myofibroblasts derived in fibrotic kidneys (upregulated and downregulated referring in WWP2-/- vs WT, Benjamini-Hochberg adjust P< 0.05)

Supplemental Figure 1

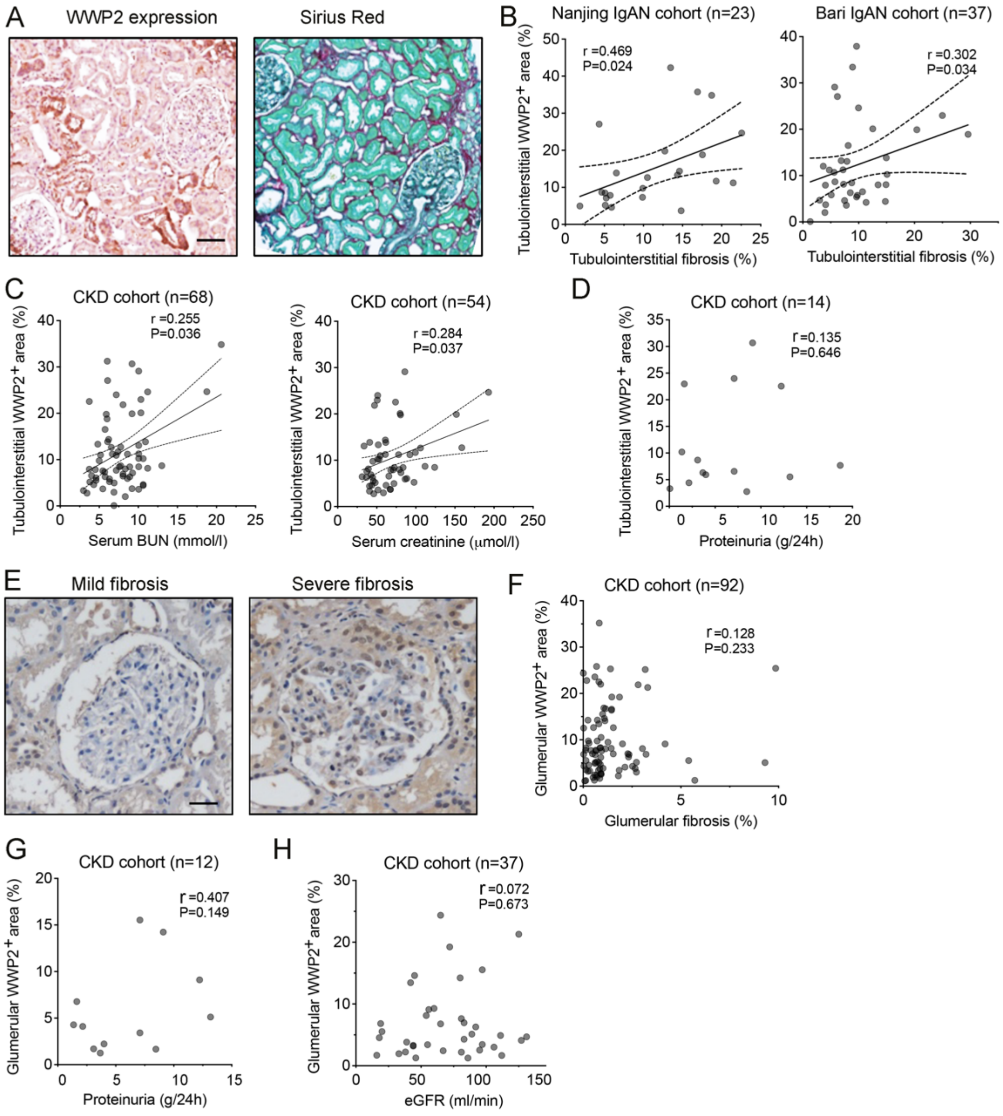

Supplemental Figure 1. Expression of WWP2 in the kidney of patients with chronic kidney disease (CKD) and UUO-mice (related to Figure 1)

(A) Representative images of WWP2 immunostaining (*left*) and Sirius Red staining (*right*) in human kidney biopsy samples, utilized for quantitative analysis for WWP2-positive area and fibrosis-positive area. Scale bars, 25 μm.

(B) Positive correlation between tubulointerstitial WWP2-positive area and fibrosis-positive area in the Nanjing IgAN cohort (n=23, *P*=0.024, *r*=0.469; *left*) and the Bari IgAN cohort (n=37, *P*=0.034, *r*=0.302; *right*), which was determined by immunostaining and Sirius Red staining in Supplemental Figure 1A using ImageScope. Data points represent measurements of individual section obtained from each patient with CKD.

(C) Positive correlation of tubulointerstitial WWP2-positive area with serum BUN levels (n=68, *P*=0.036, *r*=0.255; *left*) and with serum creatinine levels (n=54, *P*=0.037, *r*=0.284; right) in patients with CKD (multi-centre cohort).

(D) Correlation of tubulointerstitial WWP2-positive area with proteinuria levels in a subgroup of patients with CKD (n=14, *P*=0.646, *r*=0.135).

(E) Representative immunostaining images of WWP2 in glomerular area from human renal biopsy samples, illustrating mild and severe glomerular fibrosis, respectively (110 patients with CKD recorded from Bari cohort). Scale bars, 25 µm.

(F-H) Correlation of glomerular WWP2-positive area with glomerular fibrosis-positive area (n=92, *P*=0.233, *r*=0.128; panel F), proteinuria levels (n=12, *P*=0.233, *r*=0.128; panel G) and eGFR levels (n=37, *P*=0.673, *r*=0.072; panel H) in patients with CKD from Bari cohort.

Supplemental Figure 2

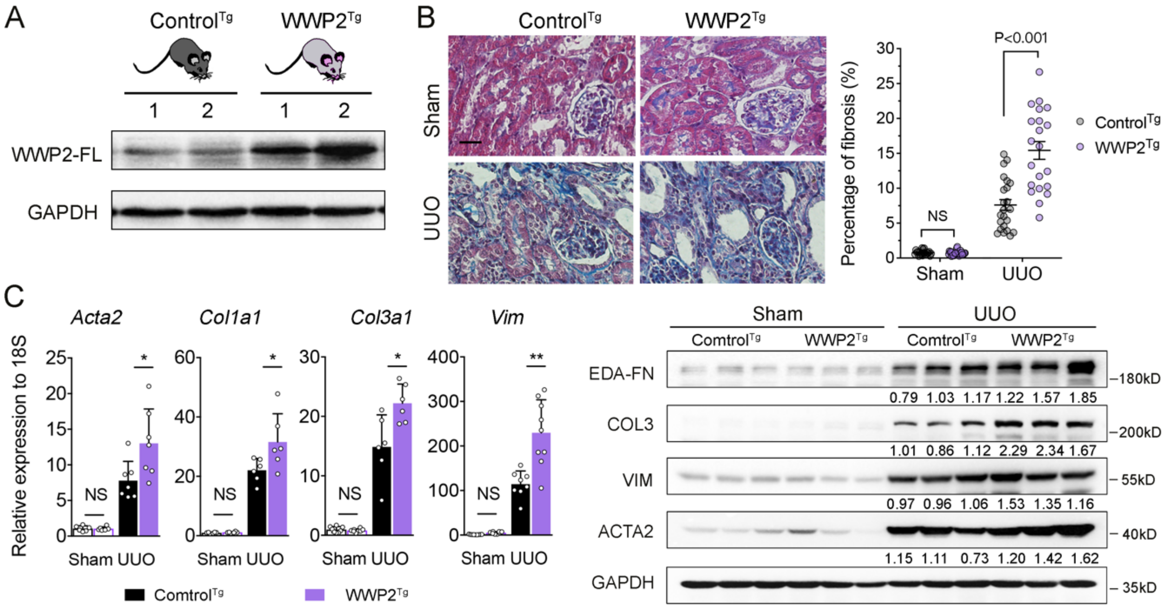

Supplemental Figure 2. WWP2 overexpression exacerbates renal fibrosis *in vivo* (related to Figure 1)

(A) Representative western blot showing the levels of WWP2 in kidney tissue from Control^{Tg} and WWP2^{Tg} mice. Control^{Tg} and WWP2^{Tg} refer to mice injected with plasmids containing control and WWP2 DNA, respectively (see Methods for details).

(B) Representative images of Masson's trichrome staining in kidney tissue sections from Sham and UUO-treated mice (10 days), with WT and WWP2$^{-/-}$ genotypes (*left*, scale bar, 30 μm), followed by quantitative analysis (*right*). Each dot indicates the average fibrosis-positive area from the section per mouse, and five non-overlapping fields were taken. Values are reported as mean ± SEM. P-values calculated by two-tailed Mann-Whitney U test.

(C) The expression of ECM genes in kidney tissues from UUO and control mice (n=6-9, each group). *Left*, expression changes were determined with RT-qPCR; *right*, representative western blot for protein levels analysis. The values are reported as mean ± SD. P-values calculated by two-tailed Mann-Whitney U test.

Supplemental Figure 3

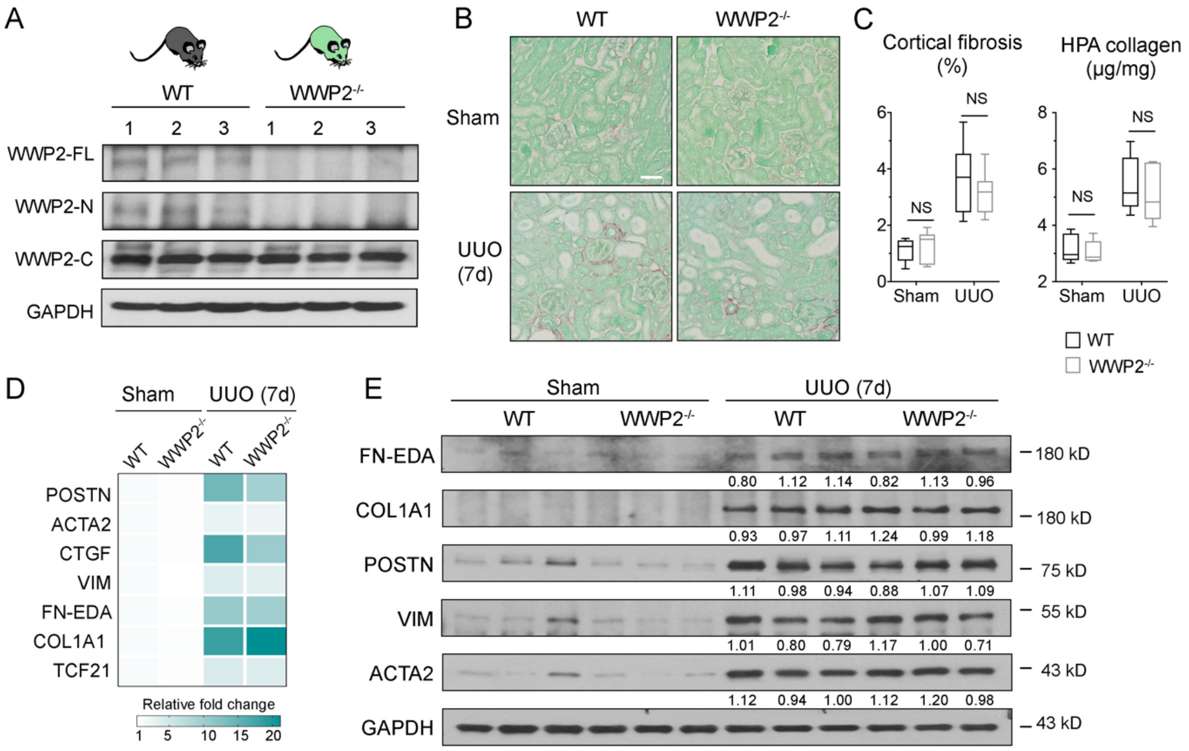

Supplemental Figure 3. Effect of WWP2 deficiency on renal fibrosis at 7 days of UUO (related to Figure 2)

(A) Representative western blot showing the levels of WWP2 in kidneys from WT and WWP2$^{-/-}$ mice. WWP2$^{-/-}$ kidneys preserve WWP2 C-terminal isoform, while lack the WWP2-full length (FL) and -N isoforms.

(B) Representative Sirius Red images of WT and WWP2$^{-/-}$ mouse kidneys following UUO model for 7 days. (n=6 images recorded for each condition). Scale bars, 10 µm.

(C) Quantitative analysis of cortical fibrosis-positive area (left, %) and HPA collage levels (right, µg/mg) WT and WWP2$^{-/-}$ mouse kidneys following UUO model for 7 days. The data values are summarized using box-and-whisker plots (n=6). P-values were calculated by two-tailed Mann-Whitney U test. NS, $P>0.05$.

(D) Heatmap showing of mRNA expression of ECM genes, determined with RT-qPCR, in kidney from WT and WWP2$^{-/-}$ mouse following UUO model for 7 days. For each gene, relative fold change was calculated with respect to Sham WT and averaged across biological replicates (n=6, each group). No statistically significant differences (P<0.05) were detected between WT and WWP2$^{-/-}$ groups.

(E) Representative western blot for ECM proteins in UUO kidneys from WT and WWP2$^{-/-}$ mouse kidneys following UUO model for 7 days. No statistically significant differences (P<0.05) were detected between WT and WWP2$^{-/-}$ groups.

Supplemental Figure 4

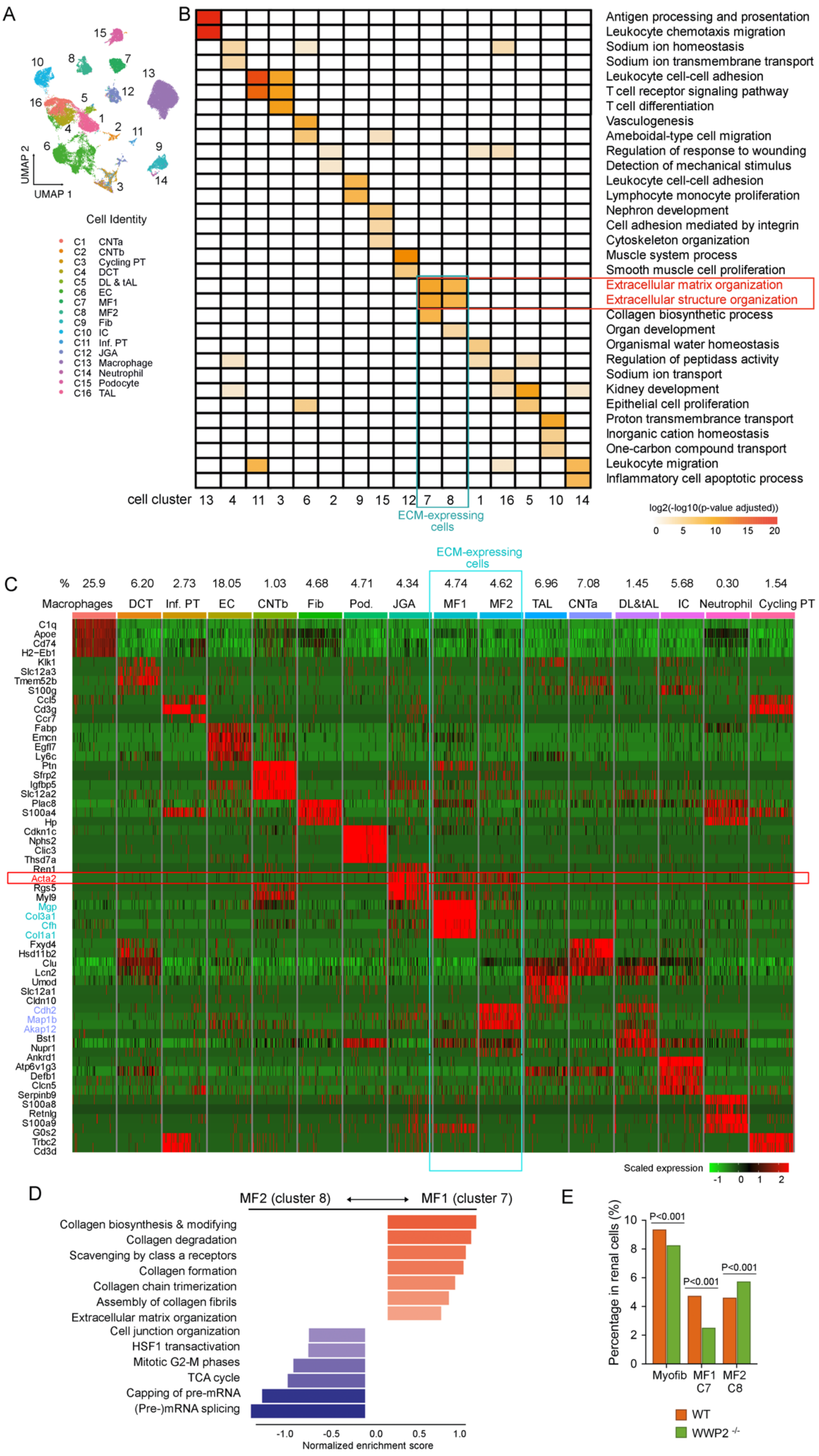

Supplemental Figure 4. Single-cell transcriptomics landscape of kidney cells derived from UUO-mice (related to Figure 3)

(A) Uniform Manifold Approximation and Projection (UMAP) representation of 74,585 kidney cells profiled in WT (n=2) and WWP2$^{-/-}$ (n=2) UUO-mice, showing 16 major cell types. CNT: connecting tubule; PT: proximal tubule; DCT: distal convoluted tubule; DL & tAL: descending limb and thin ascending limb; EC: endothelial cells; MF: myofibroblasts; Fib: fibroblasts; IC: intercalated cells; Inf. PT: inflammation proximal tubule; JGA: juxtaglomerular cells; TAL: thick ascending limb. Top 4 marker genes are reported in panel C.

(B) Heatmap of enriched pathways identified in each renal cell-type cluster. Only the top 2 significant terms are shown, and the significance of enrichment is represented by $\log_2(-\log_{10}$(adjusted p-value)). Pathways involved in ECM process are highlighted (red box) and were identified in the ECM-expressing clusters (cluster 7 and 8, light blue box).

(C) Heatmap of the scaled gene expression of top 4 marker genes for each cell cluster (panel A), based on average $\log_2$ fold change (FC) in gene expression. The marker gene ACTA-2 is highlighted (red box), which is detected in JGA cluster and in the ECM-expressing cluster 7 and 8 (light blue box).

(D) Reactome pathways enriched in myofibroblasts from UUO kidneys. These pathways are identified in the differentially expressed genes between MF1 and MF2, i.e., cluster 7 and 8, respectively, by GSEA (false discovery rate (FDR)<0.05). Positive normalized enrichment score indicates pathway upregulation in MF1 compared with MF2.

(E) Box plots showing the proportions (%) of myofibroblasts: MF1 (cluster 7, C7) and MF2 (cluster 8, C8) cells based on single cell analysis in both WT and WWP2$^{-/-}$ kidneys after UUO treatment (14 days). For each cell subset, P-value for significance of difference in cell proportions was derived by χ^2 test.

Supplemental Figure 5

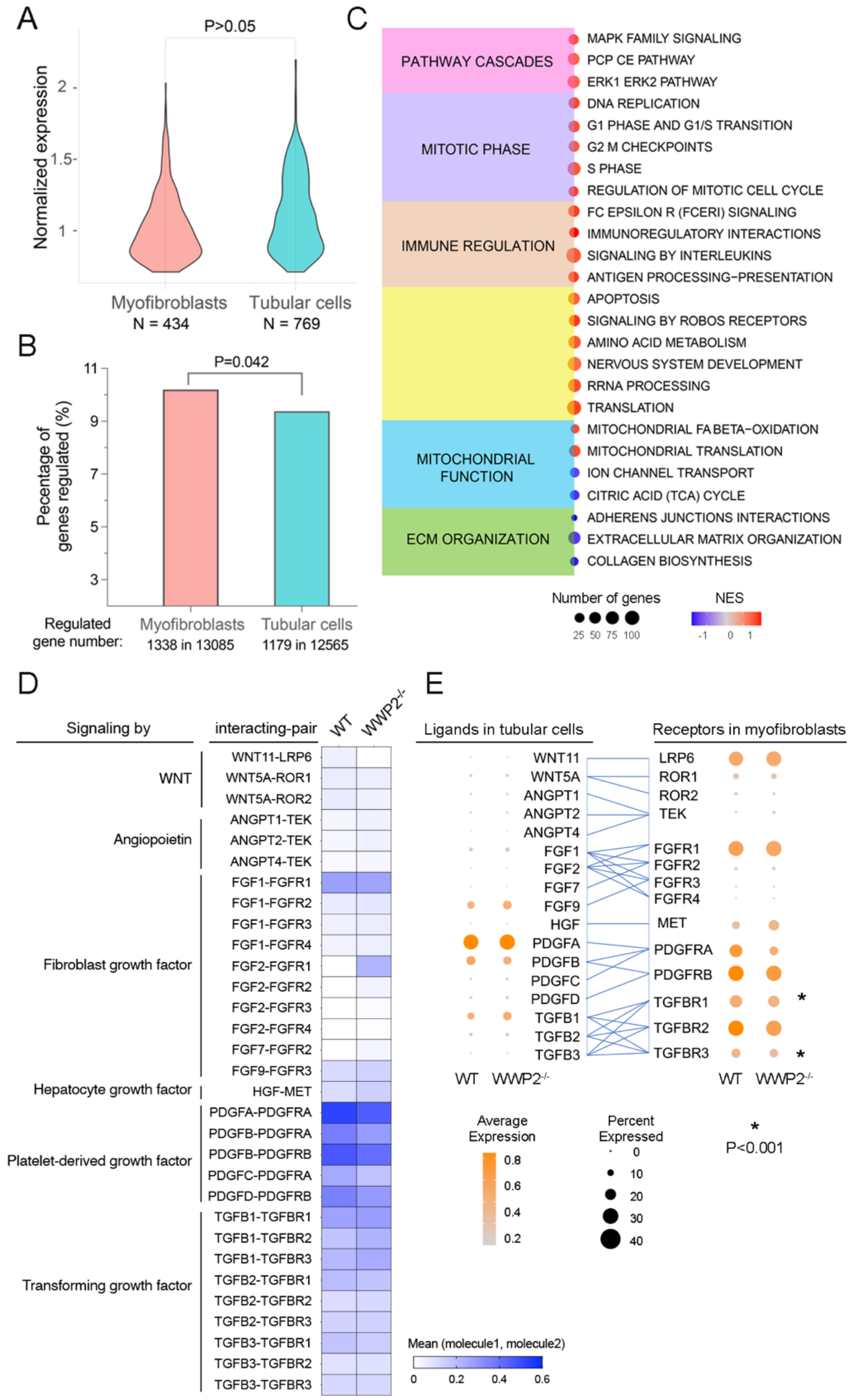

Supplemental Figure 5. Regulatory function of WWP2 in myofibroblasts and tubular cells derived from fibrotic kidneys

(A-B) Normalized expression of WWP2 (A) and the percentage of genes regulated by WWP2 (B) in renal myofibroblasts and tubular cells.

(C) Reactome pathways enriched in tubular cells and significantly different between WT and WWP2$^{-/-}$ UUO kidneys by Gene Set Enrichment Analysis (GSEA) (false discovery rate (FDR) <0.05). NES,

normalized enrichment score, where a positive NES indicate upregulation in WWP2$^{-/-}$ compared with WT myofibroblasts.

(D) Activity of previously reported chemokine ligand-receptor pairs (molecule 1-2 pairs) in tubular cells (ligands) and myofibroblasts (receptors) derived from WT and WWP2$^{-/-}$ UUO kidneys. Mean expression of molecule 1-2 pairs is reported as color intensity.

(E) Differential expression analysis (WWP2$^{-/-}$ vs WT) of chemokine ligands (in tubular cells) and receptors (in myofibroblasts). For each chemokine ligand and receptor, we report the percent of expressed cells and average mRNA expression. Significantly different interactions: *, Benjamini-Hochberg adjusted P<0.001.

Supplemental Figure 6

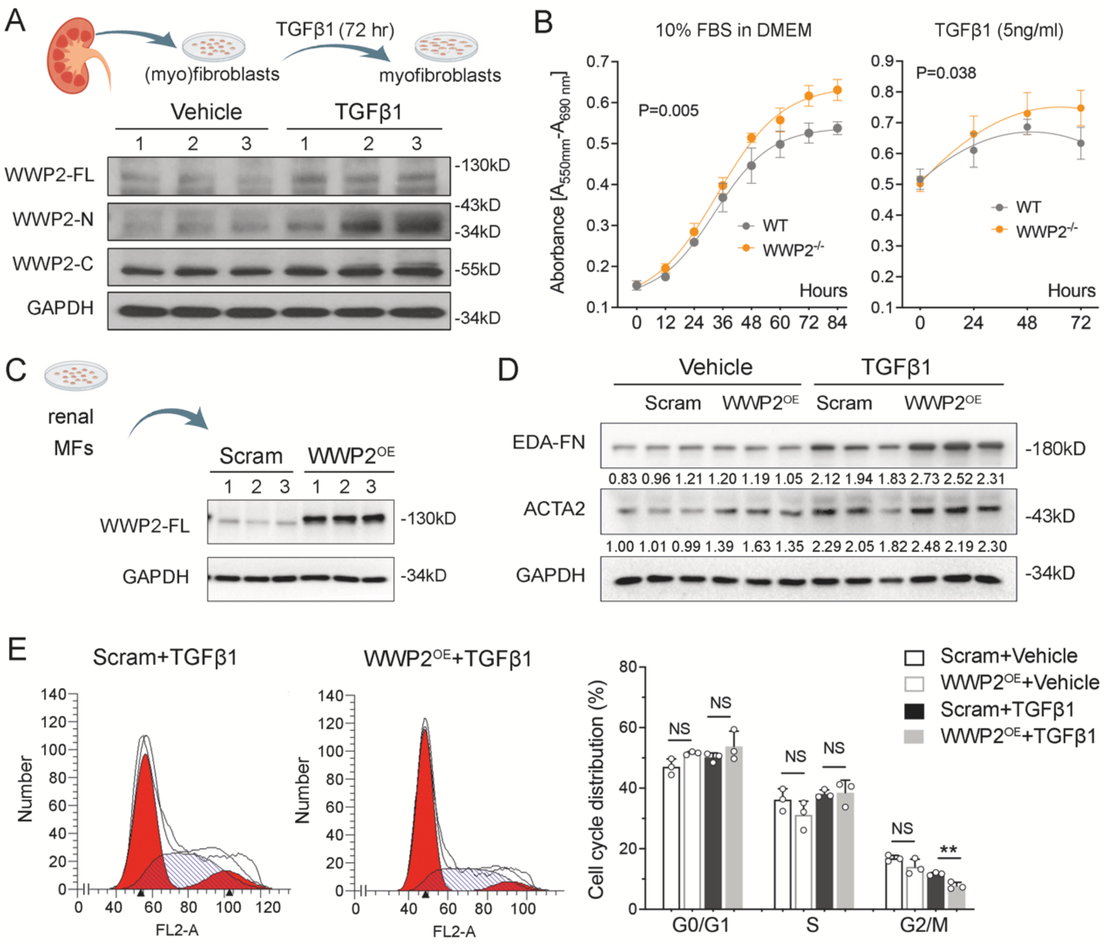

Supplemental Figure 6. WWP2 overexpression mediates cellular proliferation and activation in renal myofibroblasts *in vitro* (related to Figure 4)

(A) Representative western blot showing the levels of WWP2 isoforms in primary cultured renal myofibroblasts upon TGFβ1 stimulation (5ng/ml, 72h).

(B) Proliferation of primary cultured renal myofibroblasts was assessed by MTT assay. Both WT and WWP2$^{-/-}$ myofibroblasts were cultured with 10% FBS in DMEM (*left*, P1 cells; n=3 for each group) and treated with TGFβ1 (5ng/ml) (*right*, P2 cells; n=6 for each group). P-value for statistical significance is calculated by two-way repeated measures ANOVA; data points are reported as mean ± SD.

(C) Representative western blot showing the levels of WWP2 in primary cultured renal myofibroblasts that were transfected with WWP2 overexpression (WWP2^{OE}) or scrambled (Scram) DNAs.

(D) Representative western blot showing ECM protein levels in cultured WWP2^{OE} and Scram myofibroblasts with or without TGFβ1 stimulation (5ng/ml, 72h).

(E) *Left:* representative graph of cell cycle in cultured WWP2^{OE} and Scram myofibroblasts using flow cytometry (TGFβ1 stimulation (5ng/ml, 72h)). *Right*: quantification of cell cycle phases at G0/G1, S and G2/M in cultured WWP2^{OE} and Scram myofibroblasts (n = 3, i.e., from 3 independent experiments). Values are reported as mean ± SEM.

Supplemental Figure 7

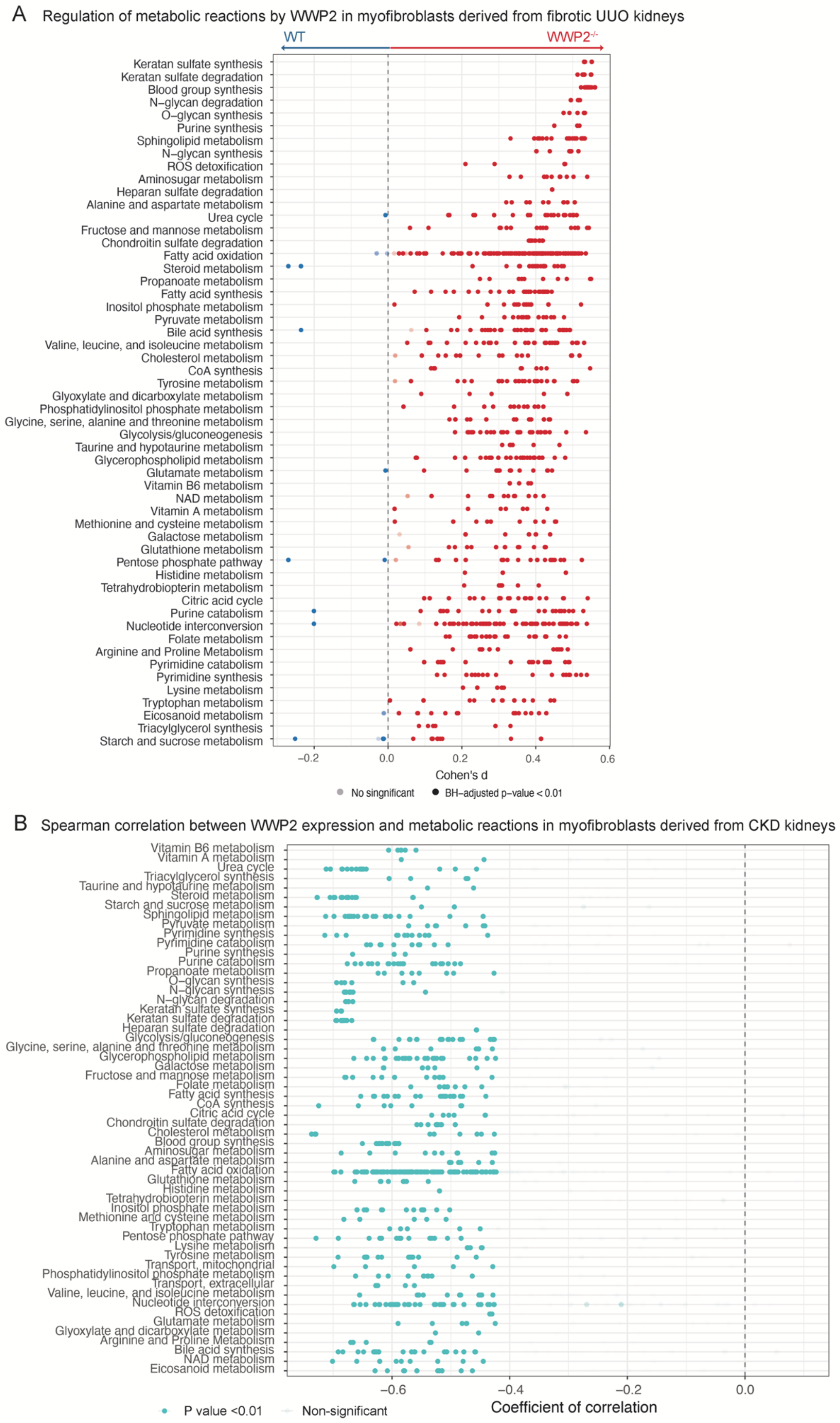

Supplemental Figure 7. Compass-based exploration of metabolic profile (related to Figure 5)

(A) Compass-score differential activity test from ECM-expressing myofibroblasts derived from WT and WWP2$^{-/-}$ UUO kidneys. Same analysis as shown in Figure 5A, but showing all metabolic reactions. BH-adjusted P-value, P-value corrected for multiple testing using the Benjamini-Hochberg method. For each metabolic reaction, the effect size of the change associated with WWP2 deficiency was estimated by Cohen's D (x-axis). The metabolic reactions upregulated in WWP2 deficient mouse kidney are indicated in red.

(B) Spearman correlation between Compass scores and the expression of WWP2 in renal myofibroblasts derived 5 CKD patients [1]. Same analysis as shown in Figure 5B, but showing all metabolic reactions. The nonsignificant correlations are shown in light grey. The metabolic reactions that are negatively correlated (P<0.01) with WWP2 expression in myofibroblasts are indicated in light blue.

Supplemental Figure 8

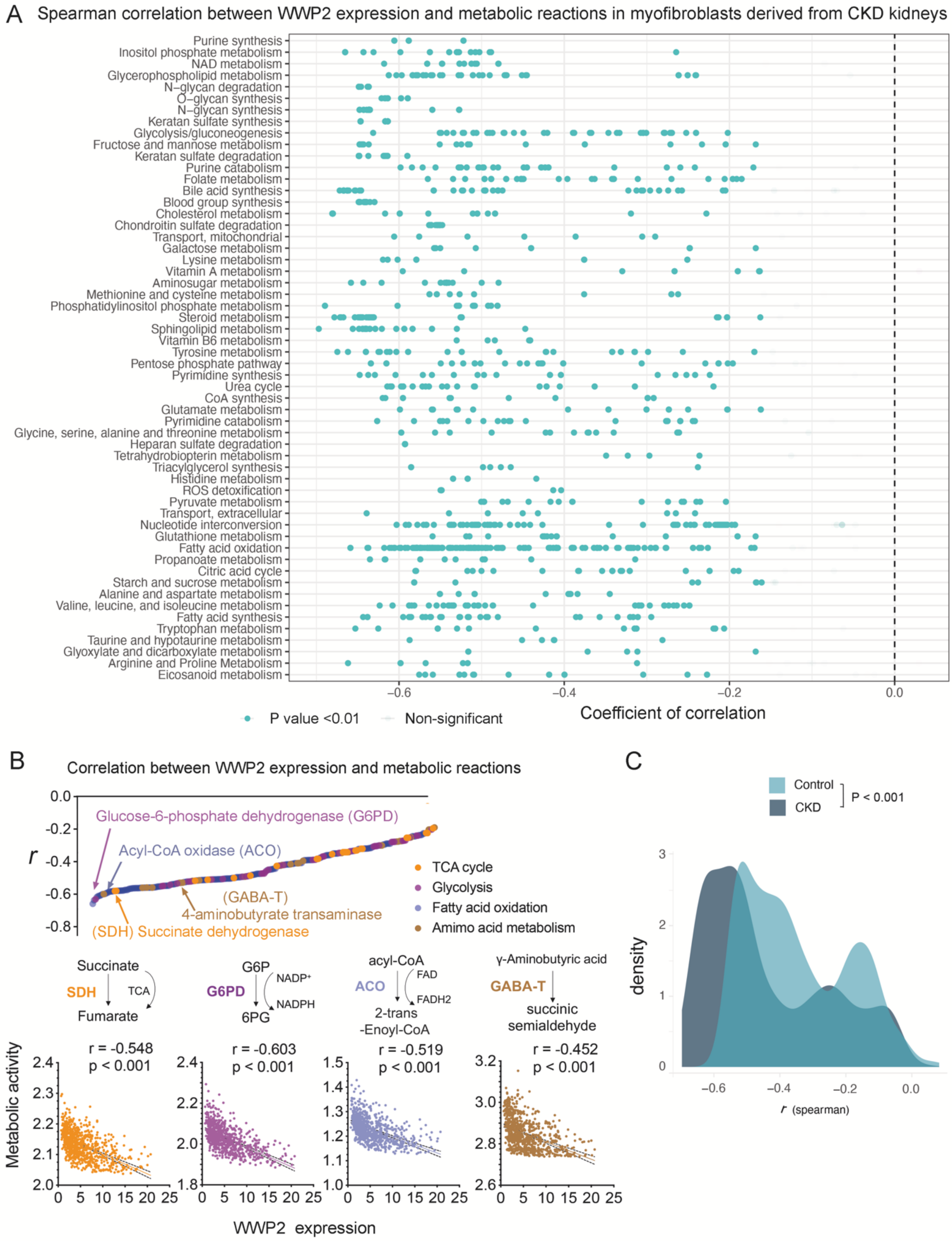

Supplemental Figure 8. Compass-based exploration of metabolic reactions regulated by WWP2 in another CKD cohort (related to Figure 5)

(A) Spearman correlation between Compass scores and the expression of WWP2 in renal myofibroblasts derived 10 patients with CKD [2]. Same analysis as shown in Supplemental Figure 6B (in 5 CKD patients) and nonsignificant correlations shown in light grey. The metabolic reactions that are negatively correlated (P<0.01) with WWP2 expression in myofibroblasts are indicated in light blue.

(B) Spearman correlation of Compass scores with WWP2 expression in renal myofibroblasts from 10 CKD patients. *Upper*: The significant Spearman correlation between Compass scores and the expression le vels of WWP2 in renal myofibroblasts. The color coding represents different metabolic pathways. *Lower*: Spearman correlation analysis for 4 key metabolic reactions. The whole set of metabolic reactions was showed in supplemental Figure 7A.

(C) Histogram showing the distribution of Spearman correlations between WWP2 expression and metabolic actions in renal myofibroblasts derived from 12 controls and 10 CKD patients, showing that the correlation (association) is significantly stronger (P<0.001) in CKD compared with control myofibroblasts. P-value was calculated using two-tailed Wilcoxon rank-sum test.

Supplemental Figure 9

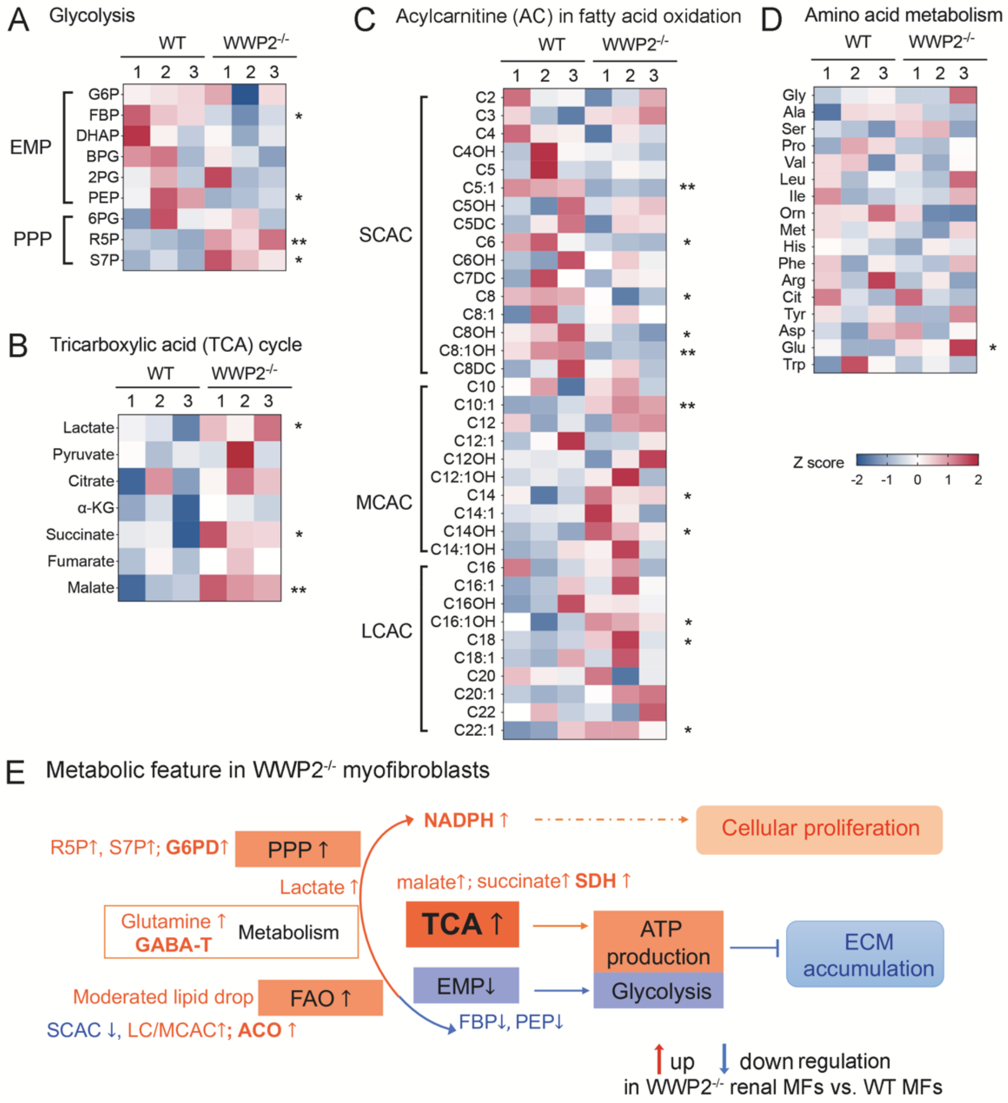

Supplemental Figure 9. Profile of metabolites measured in cultured renal myofibroblasts derived from WT and WWP2$^{-/-}$ kidneys (related to Figure 5)

(A-D) Metabolomics profile of metabolites in cultured renal myofibroblasts (P2) derived from WT and WWP2$^{-/-}$ kidneys, arranged according to metabolic pathways: glycolysis pathway (A), TCA cycle (B), ACs in fatty acid oxidation pathway (C), and amino acid metabolism (D). Same analysis as shown in Figure 5C, but showing all metabolites (and not just significantly differentially expressed between WT and WWP2$^{-/-}$, as in the main figure). Each metabolite was presented as Z score. (E). Schematic representation illustrating the metabolic processes changed in WWP2$^{-/-}$ myofibroblasts.

EMP: Embden-Meyerhof pathway of glycolysis; PPP: pentose phosphate pathway; FBP: Fructose-1,6-bisphosphate; PEP: Phosphoenolpyruvate; R5P: Ribose-5-phosphate; S7P: Sedoheptulose-7-phosphate; G6P: Glucose-6-phosphate; DHAP: Dihydroxyacetone phosphate; BPG: Bisphosphoglycerate or 2,3-Bisphosphoglycerate; 2PG: 2-Phosphoglycerate; SCAC: small chain of acylcarnitine; LCAC: long chain of acylcarnitine; MCAC: Medium chain of acylcarnitine; α-KG, a-ketoglutarate; Gly: glycine; Ala: alanine; Ser: serine; Pro: proline; Val: valine; Leu: leucine; Ile: isoleucine; Orn: ornithine; Met: methionine; His: histidine; Phe: phenylalanine; Arg: arginine; Cit: citrate; Tyr: tyrosine; Asp: aspartic acid; Glu: glutamic acid; Trp: tryptophan.

Supplemental Figure 10

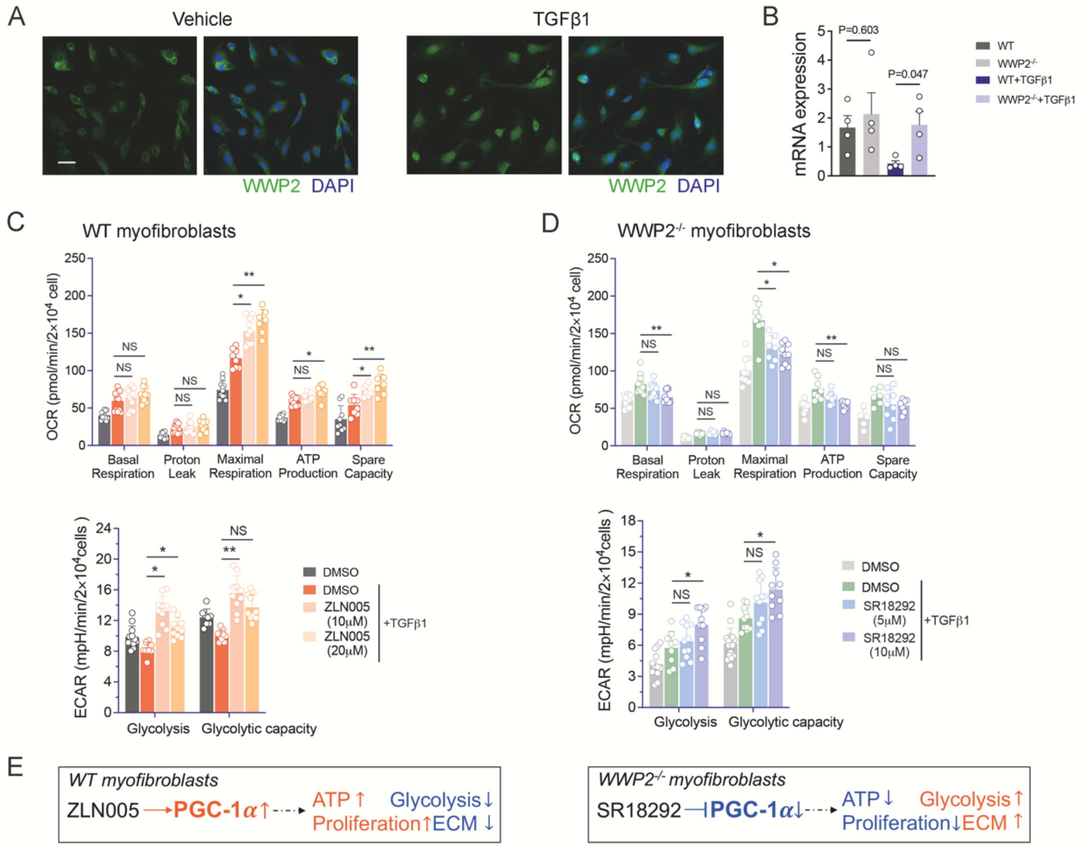

Supplemental Figure 10. Regulation of PGC-1α signalling by WWP2 (related to Figure 6)

(A) Representative immunofluorescence analysis of WWP2 in WT primary renal myofibroblasts showing nuclear localization after 24 hrs TGFβ1 stimulation (5ng/ml). Scale bar: 20 µm.

(B) Normalized gene expression levels (TPM, transcripts per million kilobase) measured by bulk-RNA seq analysis of PGC-1α in myofibroblasts derived from WT and WWP2$^{-/-}$ kidneys with or without TGFβ1 stimulation (5ng/ml, 72hrs). n=4, from independent biological replicates. Values are reported as mean ± SEM.

(C) Barplots summarizing the effects of ZLN005 (10 µM and 20 µM) on OCR based on Seahorse Mito stress assays (*upper panel*) and on ECAR based on Seahorse Glycolysis assays (*lower panel*) in cultured WT myofibroblasts. n=3 independent experiments, each containing readouts from 3-4 microplate wells (technical replicates), yielding n=9-12 data points.

(D) Barplots summarizing the effects of SR18292 (2.5 µM and 5 µM) on OCR based on Seahorse Mito stress assays (*upper panel*) and on ECAR based on Seahorse Glycolysis assays (*lower panel*) in cultured WWP2$^{-/-}$ myofibroblasts. n=3 independent experiments, each containing readouts from 3-4 microplate wells (technical replicates), yielding n=9-12 data points.

(E) Schematic summary of the cellular and metabolic phenotypes observed following PGC-1α pharmacological activation (by ZLN005) or inhibition (by SR18292) in primary cultured WT and WWP2$^{-/-}$ renal myofibroblasts, respectively.

References

1. Kuppe, C., et al., *Decoding myofibroblast origins in human kidney fibrosis.* Nature, 2021. **589**(7841): p. 281-286.
2. The results here are in whole or part based upon data generated by KPMP: DK133081, D., DK133092,DK133093, DK133095, DK1330971, DK114866, DK114908, DK133090, DK133113,DK133766, DK133768, DK114907, DK114920, DK114923, DK114933, DK114886. https://www.kpmp.org.
3. Santana-Garcia, W., et al., *RSAT 2022: regulatory sequence analysis tools.* Nucleic Acids Res, 2022. **50**(W1): p. W670-W676.
4. Aibar, S., et al., *SCENIC: single-cell regulatory network inference and clustering.* Nat Methods, 2017. **14**(11): p. 1083-1086.
5. Hammal, F., et al., *ReMap 2022: a database of Human, Mouse, Drosophila and Arabidopsis regulatory regions from an integrative analysis of DNA-binding sequencing experiments.* Nucleic Acids Res, 2022. **50**(D1): p. D316-D325.

Figure 1e

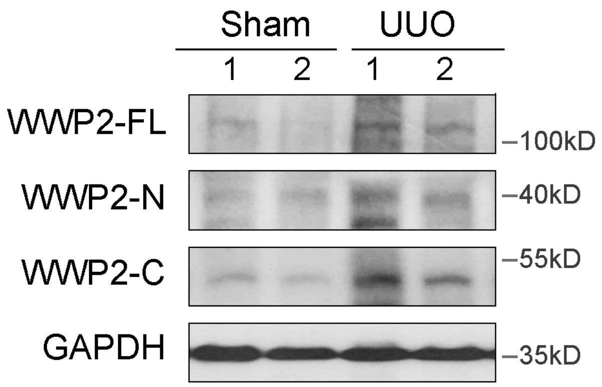

Sham
UUO
1
2
1
2
WWP2-FL
WWP2-N
WWP2-C
GAPDH
–100kD
–40kD
–55kD
–35kD

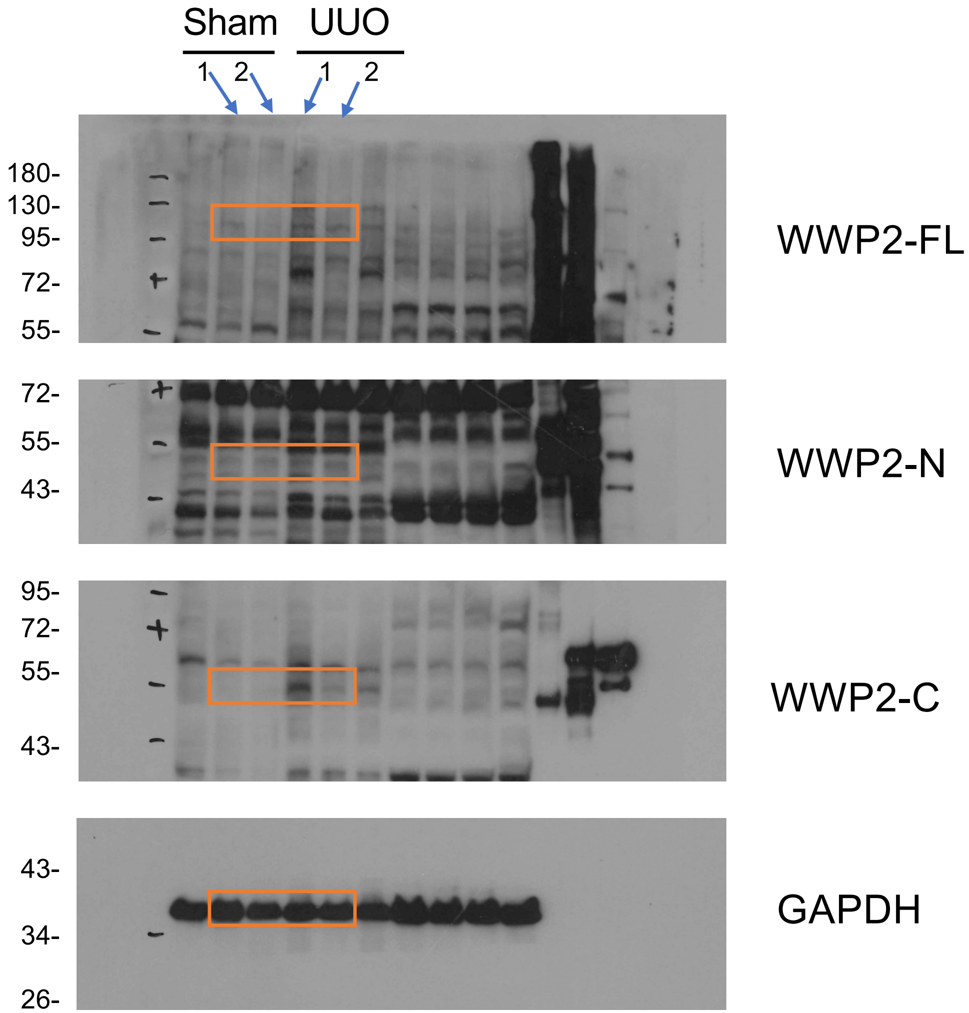

Sham
UUO
1
2
1
2
180-
130-
95-
72-
55-
WWP2-FL
72-
55-
43-
WWP2-N
95-
72-
55-
43-
WWP2-C
43-
34-
26-
GAPDH

Figure 2c

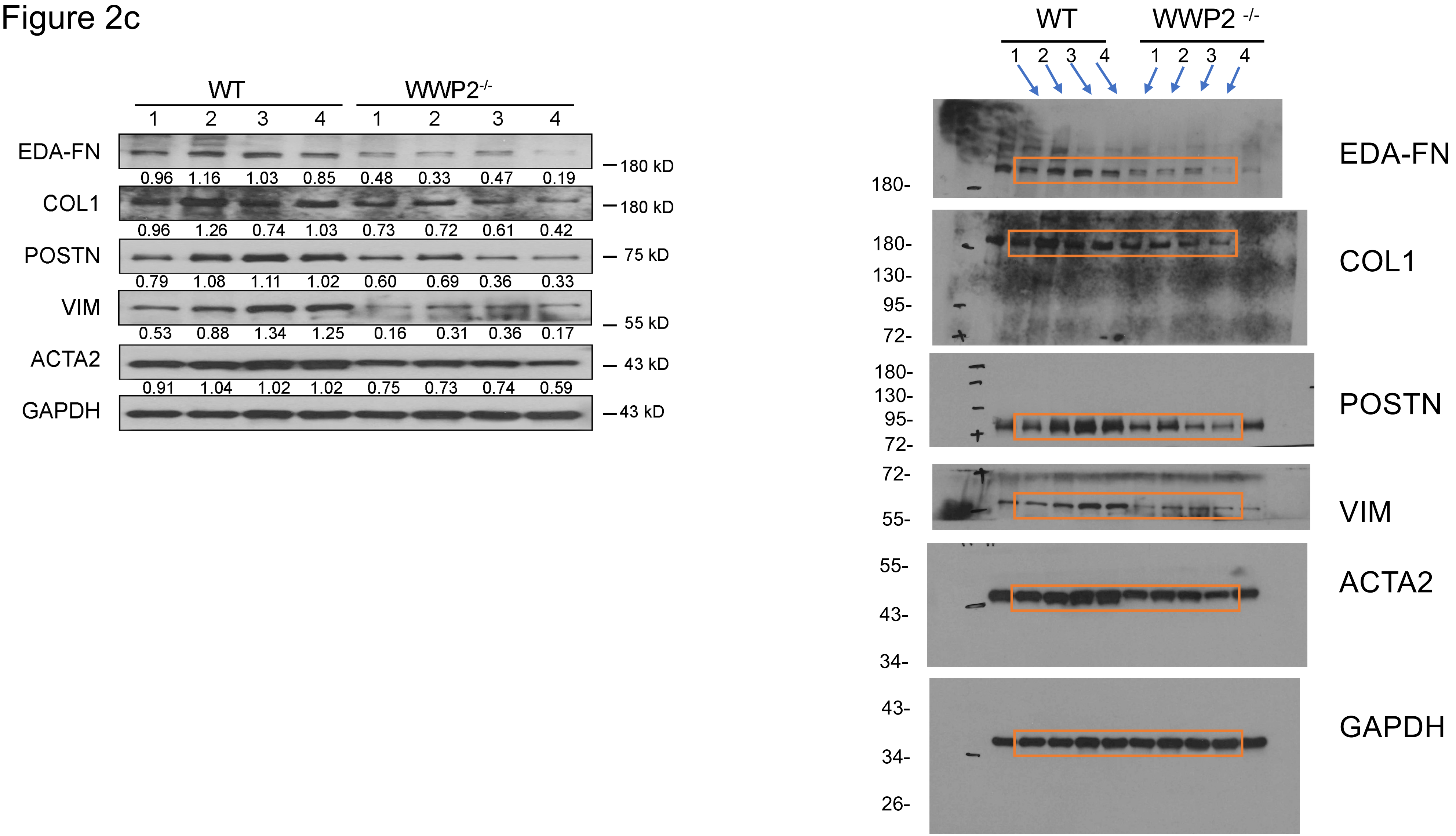
WT
WWP2-/-
1 2 3 4 1 2 3 4
EDA-FN
0.96 1.16 1.03 0.85 0.48 0.33 0.47 0.19
180 kD
COL1
0.96 1.26 0.74 1.03 0.73 0.72 0.61 0.42
180 kD
POSTN
0.79 1.08 1.11 1.02 0.60 0.69 0.36 0.33
75 kD
VIM
0.53 0.88 1.34 1.25 0.16 0.31 0.36 0.17
55 kD
ACTA2
0.91 1.04 1.02 1.02 0.75 0.73 0.74 0.59
43 kD
GAPDH
43 kD
WT
WWP2 -/-
1 2 3 4 1 2 3 4
180-
EDA-FN
180-
130-
95-
72-
COL1
180-
130-
95-
72-
POSTN
72-
55-
VIM
55-
43-
34-
ACTA2
43-
34-
26-
GAPDH

Figure 2g

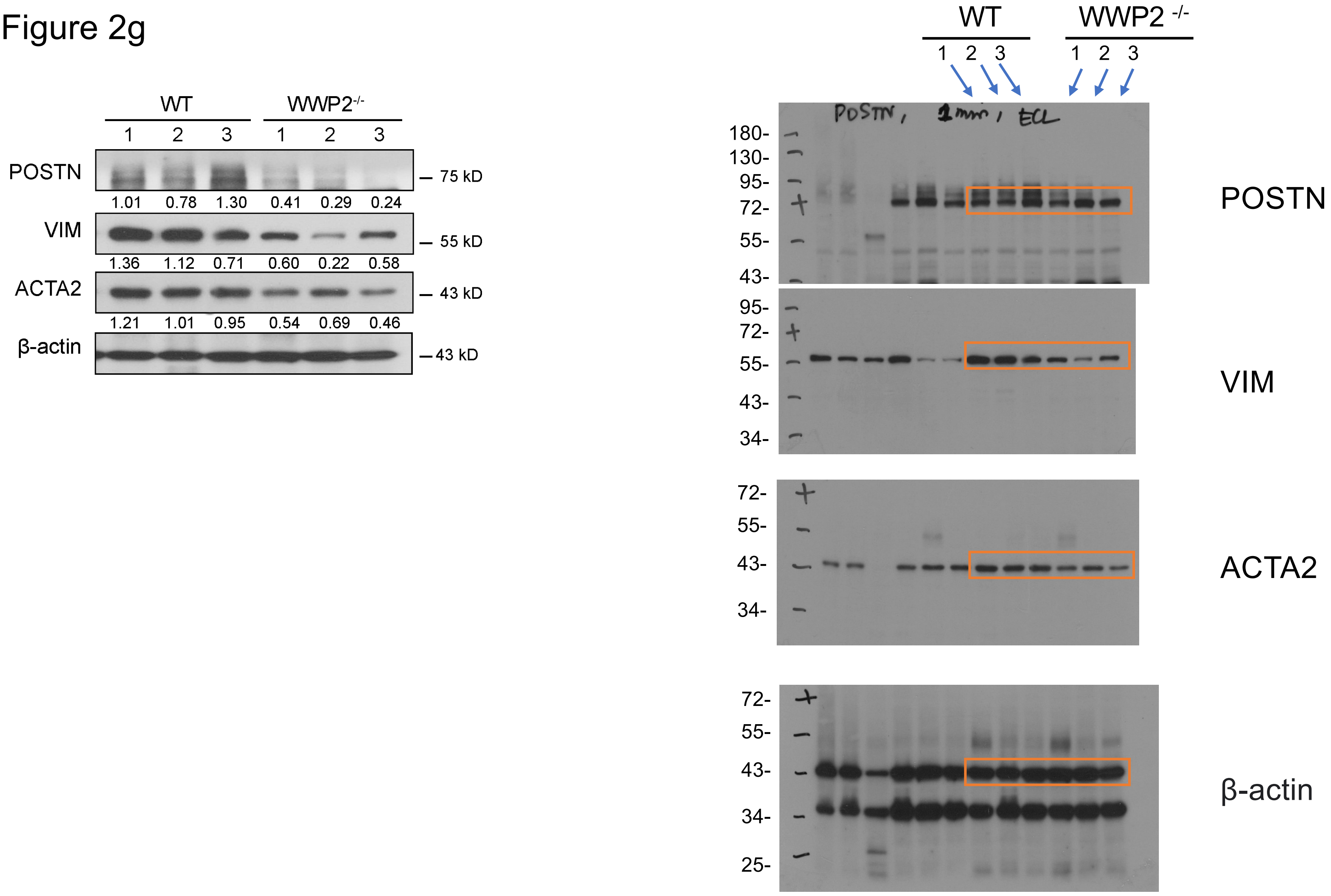
WT
WWP2-/-
1 2 3 1 2 3
POSTN
1.01 0.78 1.30 0.41 0.29 0.24
VIM
1.36 1.12 0.71 0.60 0.22 0.58
ACTA2
1.21 1.01 0.95 0.54 0.69 0.46
β-actin
75 kD
55 kD
43 kD
43 kD
WT
WWP2 -/-
1 2 3 1 2 3
180-
130-
95-
72-
55-
43-
34-
25-
POSTN
VIM
ACTA2
β-actin

Figure 4a

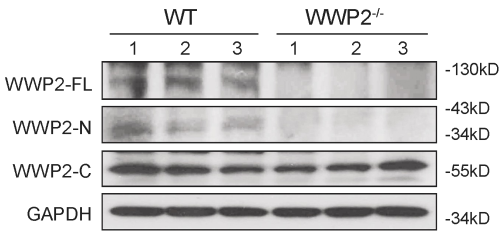
WT
WWP2-/-
1 2 3 1 2 3
WWP2-FL
-130kD
WWP2-N
-43kD
-34kD
WWP2-C
-55kD
GAPDH
-34kD

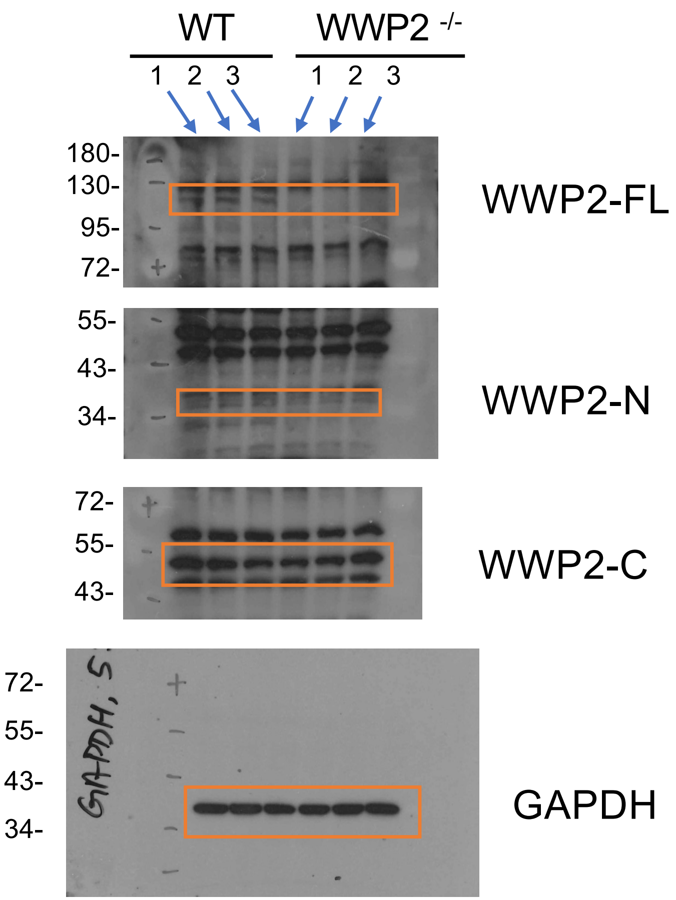
WT
WWP2 -/-
1 2 3 1 2 3
180-
130-
95-
72-
WWP2-FL
55-
43-
34-
WWP2-N
72-
55-
43-
WWP2-C
72-
55-
43-
34-
GAPDH

Figure 4d

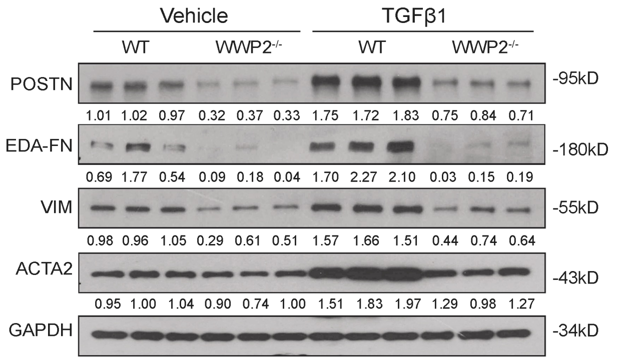

Vehicle
TGFβ1
WT
WWP2-/-
WT
WWP2-/-
POSTN
-95kD
1.01 1.02 0.97 0.32 0.37 0.33 1.75 1.72 1.83 0.75 0.84 0.71
EDA-FN
-180kD
0.69 1.77 0.54 0.09 0.18 0.04 1.70 2.27 2.10 0.03 0.15 0.19
VIM
-55kD
0.98 0.96 1.05 0.29 0.61 0.51 1.57 1.66 1.51 0.44 0.74 0.64
ACTA2
-43kD
0.95 1.00 1.04 0.90 0.74 1.00 1.51 1.83 1.97 1.29 0.98 1.27
GAPDH
-34kD

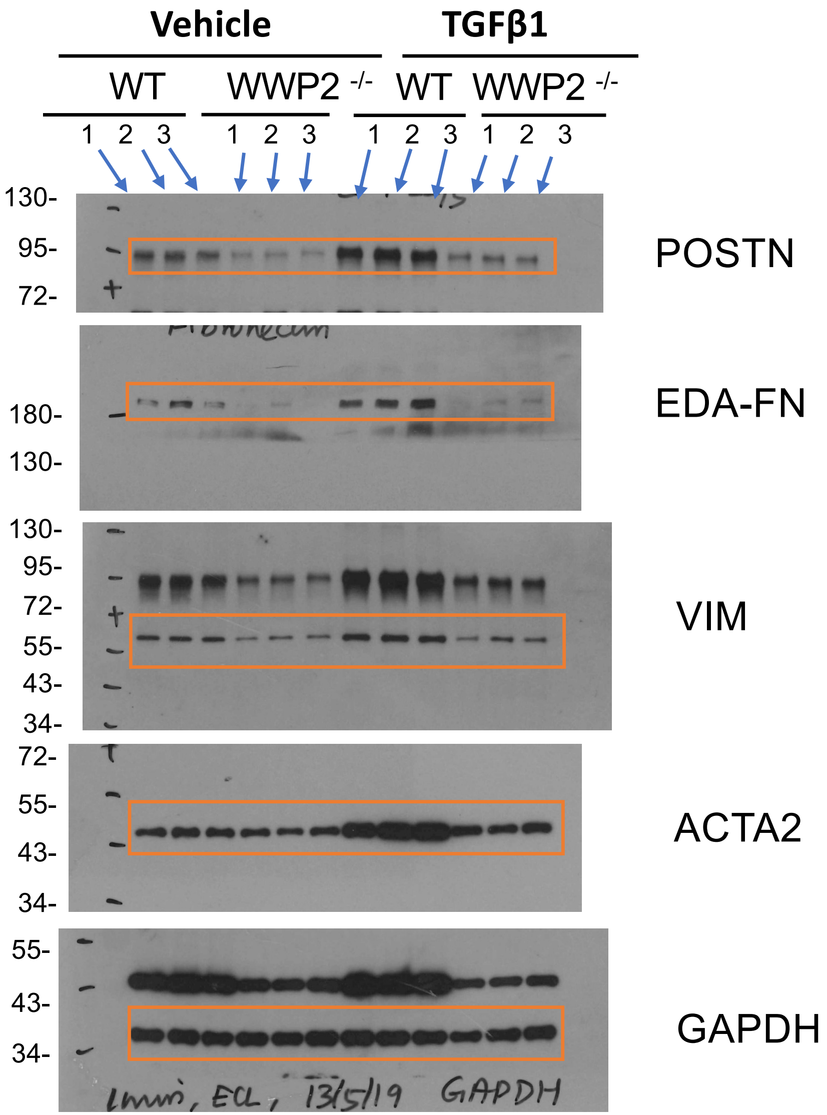

Vehicle
TGFβ1
WT
WWP2 -/-
WT
WWP2 -/-
1 2 3 1 2 3 1 2 3 1 2 3
130-
95-
72-
POSTN
180-
130-
EDA-FN
130-
95-
72-
55-
43-
34-
VIM
72-
55-
43-
34-
ACTA2
55-
43-
34-
GAPDH

Figure 4h

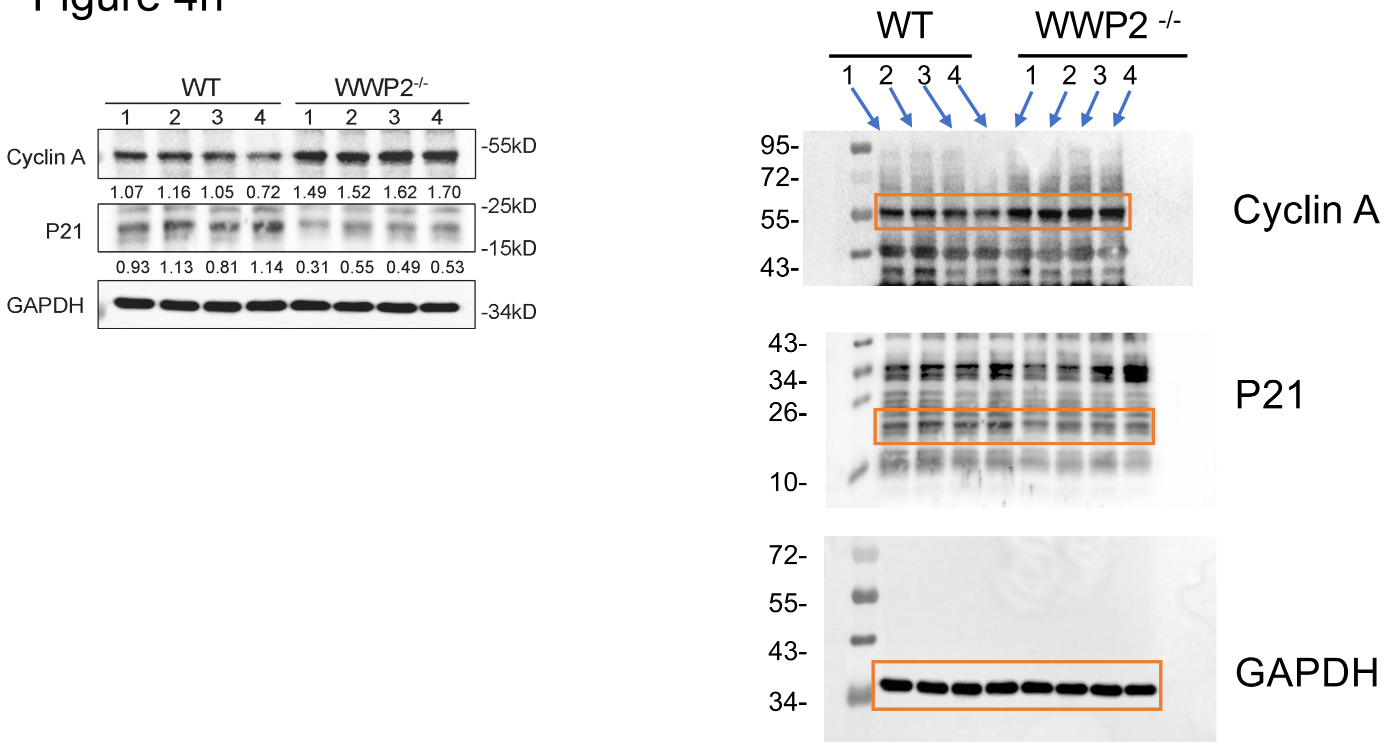
WT
WWP2-/-
1 2 3 4 1 2 3 4
Cyclin A
-55kD
1.07 1.16 1.05 0.72 1.49 1.52 1.62 1.70
-25kD
P21
-15kD
0.93 1.13 0.81 1.14 0.31 0.55 0.49 0.53
GAPDH
-34kD
WT
WWP2 -/-
1 2 3 4 1 2 3 4
95-
72-
55-
43-
Cyclin A
43-
34-
26-
10-
P21
72-
55-
43-
34-
GAPDH

Figure 6c

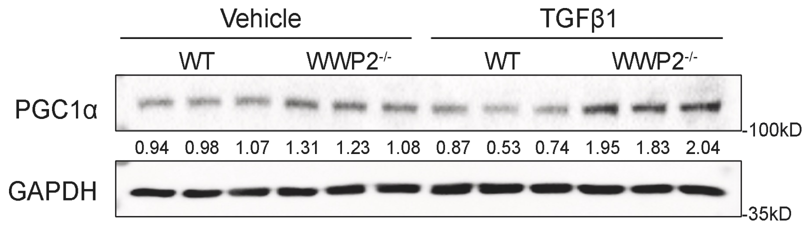

Vehicle
TGFβ1
WT
WWP2-/-
WT
WWP2-/-
PGC1α
0.94 0.98 1.07 1.31 1.23 1.08 0.87 0.53 0.74 1.95 1.83 2.04
GAPDH
100kD
35kD

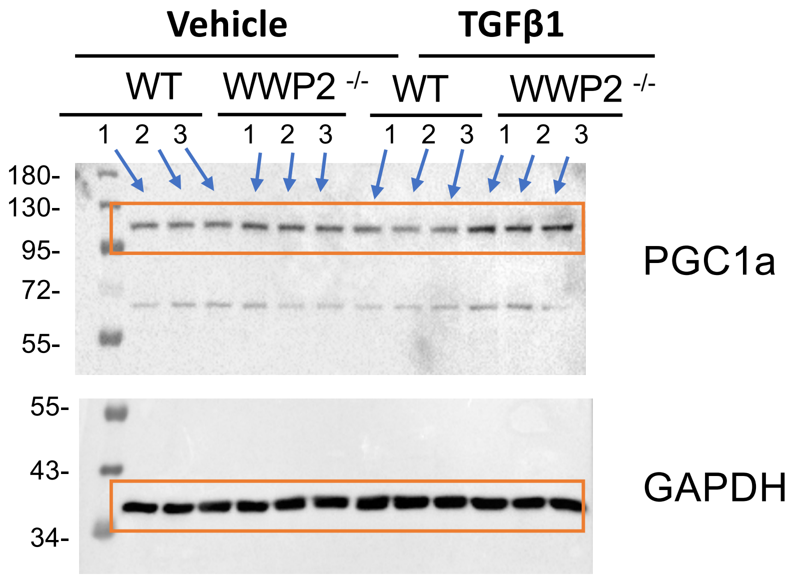

Vehicle
TGFβ1
WT
WWP2 -/-
WT
WWP2 -/-
1 2 3 1 2 3 1 2 3 1 2 3
180-
130-
95-
72-
55-
PGC1a
55-
43-
34-
GAPDH

Figure 6i

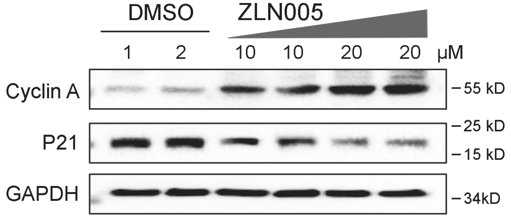

DMSO
ZLN005
1
2
10
10
20
20
μM
Cyclin A
P21
GAPDH
55 kD
25 kD
15 kD
34kD

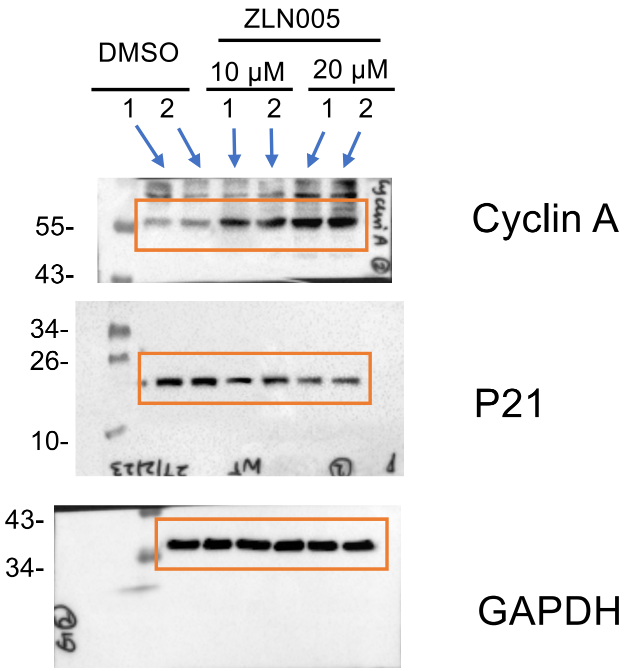

ZLN005
DMSO
10 μM
20 μM
1 2 1 2 1 2
55-
43-
Cyclin A
34-
26-
10-
P21
43-
34-
GAPDH

Figure 6j

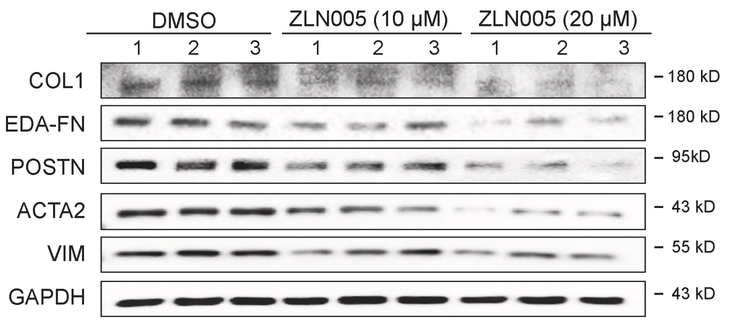

DMSO
ZLN005 (10 μM)
ZLN005 (20 μM)
1 2 3 1 2 3 1 2 3
COL1
EDA-FN
POSTN
ACTA2
VIM
GAPDH
180 kD
180 kD
95kD
43 kD
55 kD
43 kD

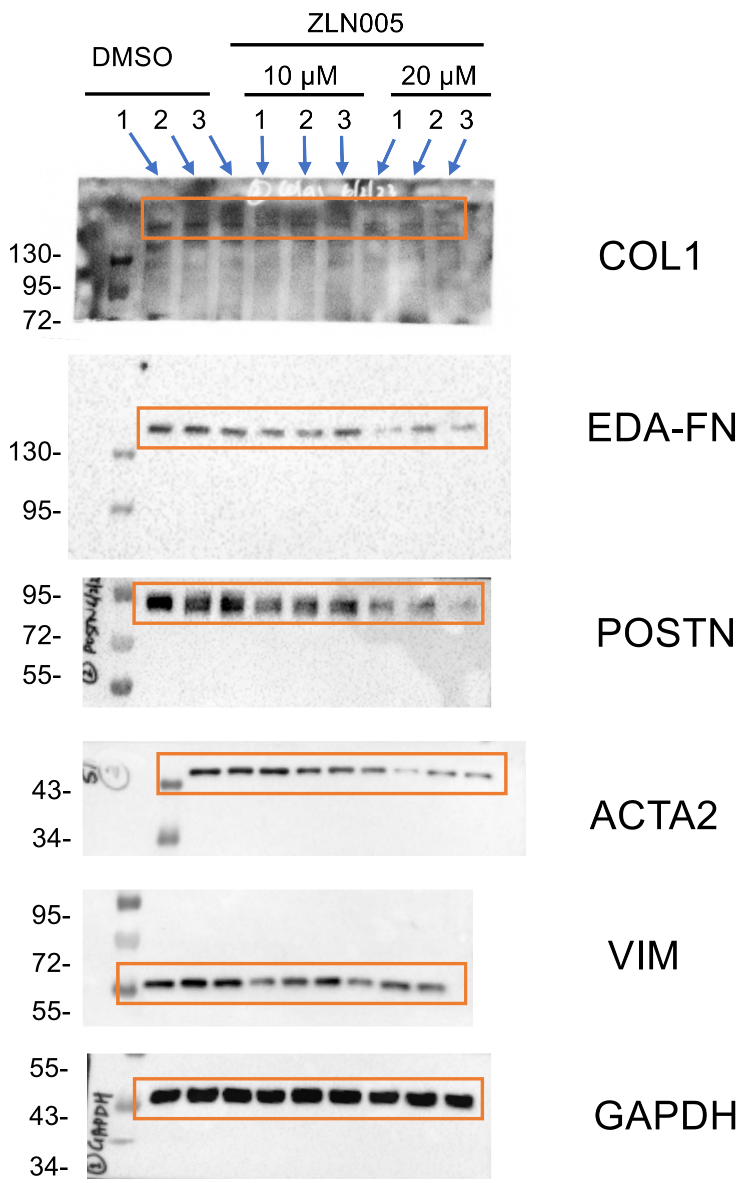

ZLN005
DMSO
10 μM
20 μM
1 2 3 1 2 3 1 2 3
130-
95-
72-
COL1
130-
95-
EDA-FN
95-
72-
55-
POSTN
43-
34-
ACTA2
95-
72-
55-
VIM
55-
43-
34-
GAPDH

Figure 6I

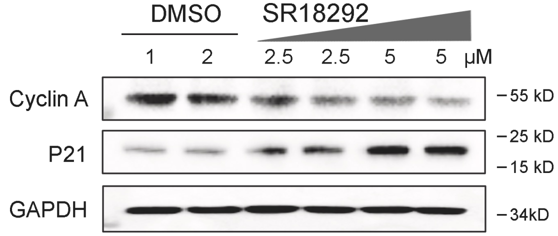
DMSO
SR18292
1
2
2.5
2.5
5
5
μM
Cyclin A
P21
GAPDH
55 kD
25 kD
15 kD
34kD

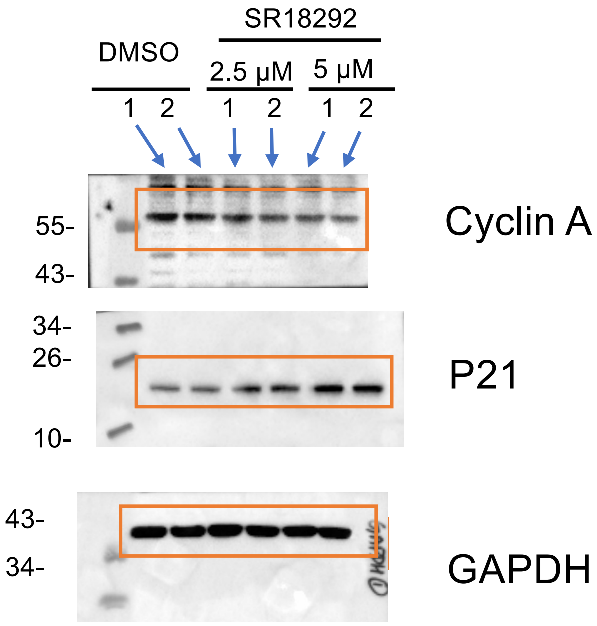
SR18292
DMSO
2.5 μM
5 μM
1 2 1 2 1 2
55-
43-
34-
26-
10-
43-
34-
Cyclin A
P21
GAPDH

Figure 6m

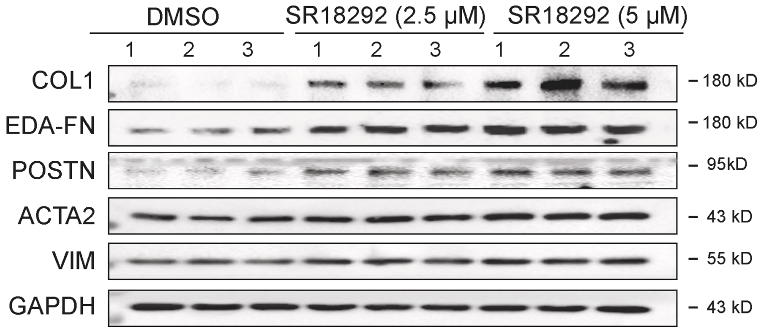

DMSO
SR18292 (2.5 μM)
SR18292 (5 μM)
1 2 3 1 2 3 1 2 3
COL1
EDA-FN
POSTN
ACTA2
VIM
GAPDH
180 kD
180 kD
95kD
43 kD
55 kD
43 kD

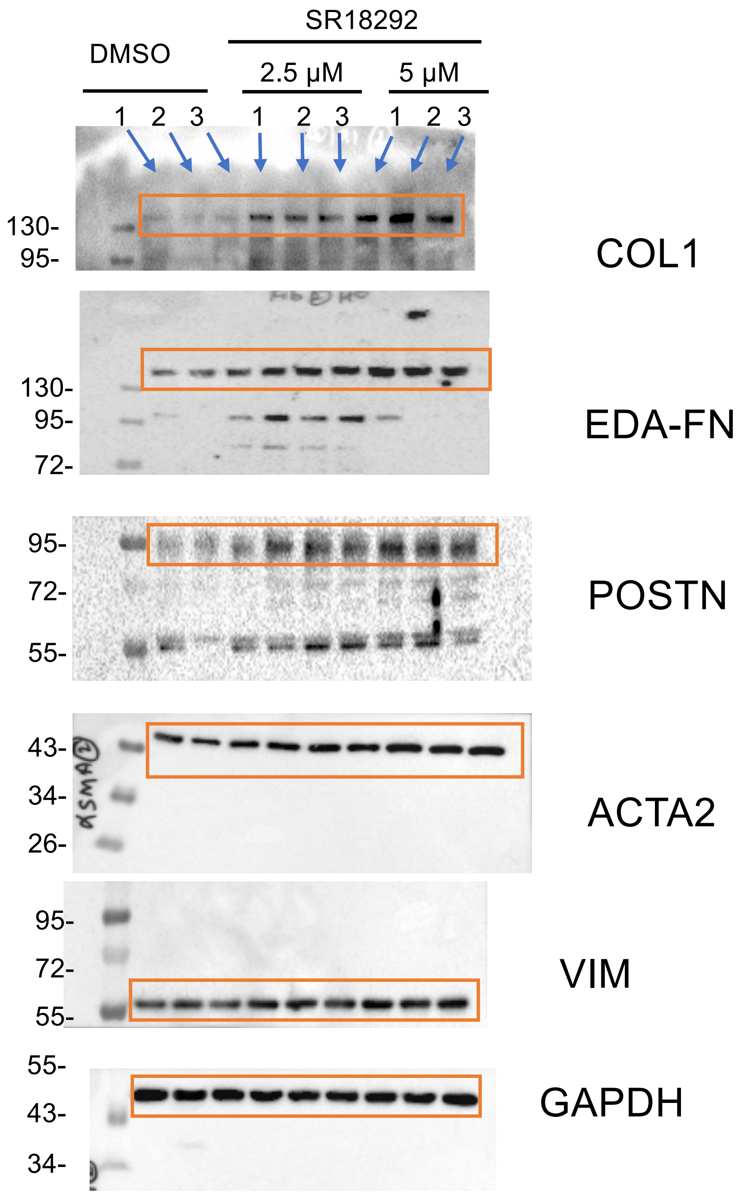

SR18292
DMSO
2.5 μM
5 μM
1 2 3 1 2 3 1 2 3
130-
95-
COL1
130-
95-
72-
EDA-FN
95-
72-
55-
POSTN
43-
34-
26-
ACTA2
95-
72-
55-
VIM
55-
43-
34-
GAPDH

Supplementary Figure 2a

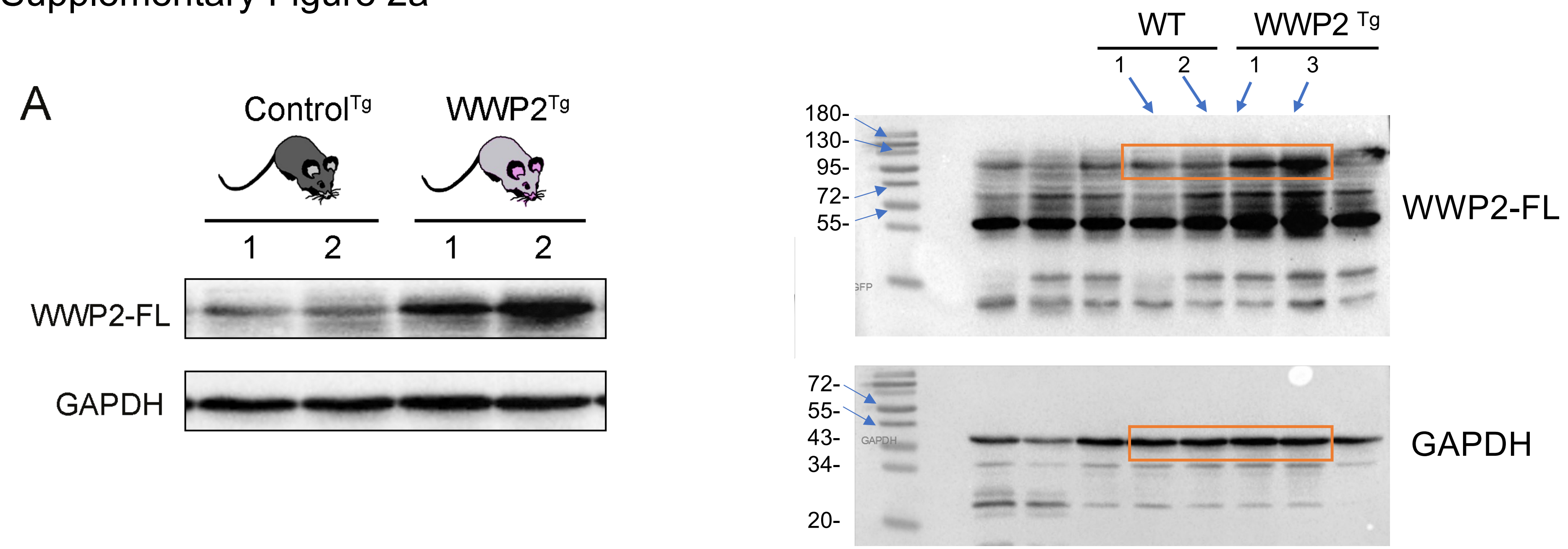

Supplementary Figure 2c

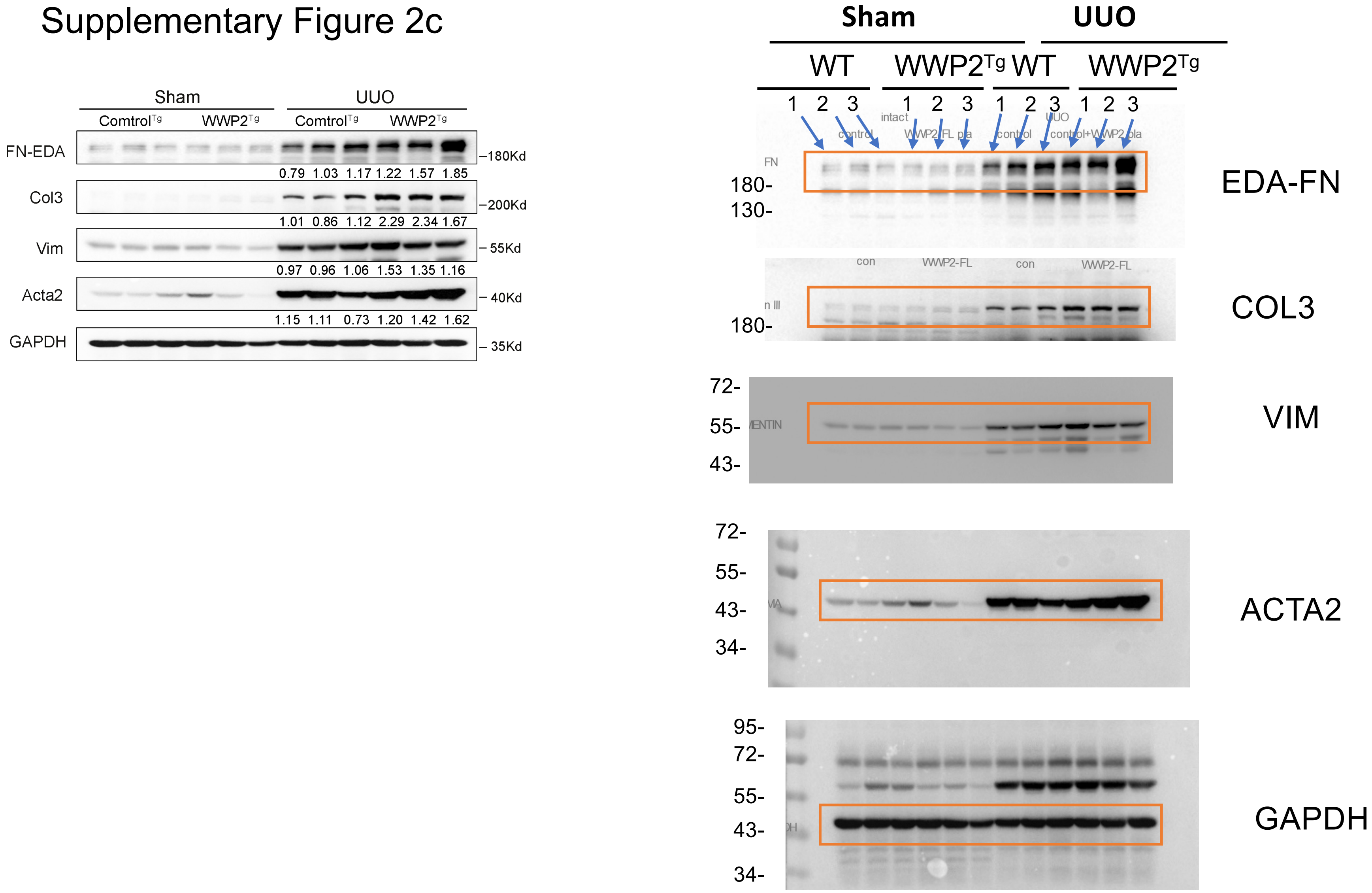

Supplementary Figure 3a

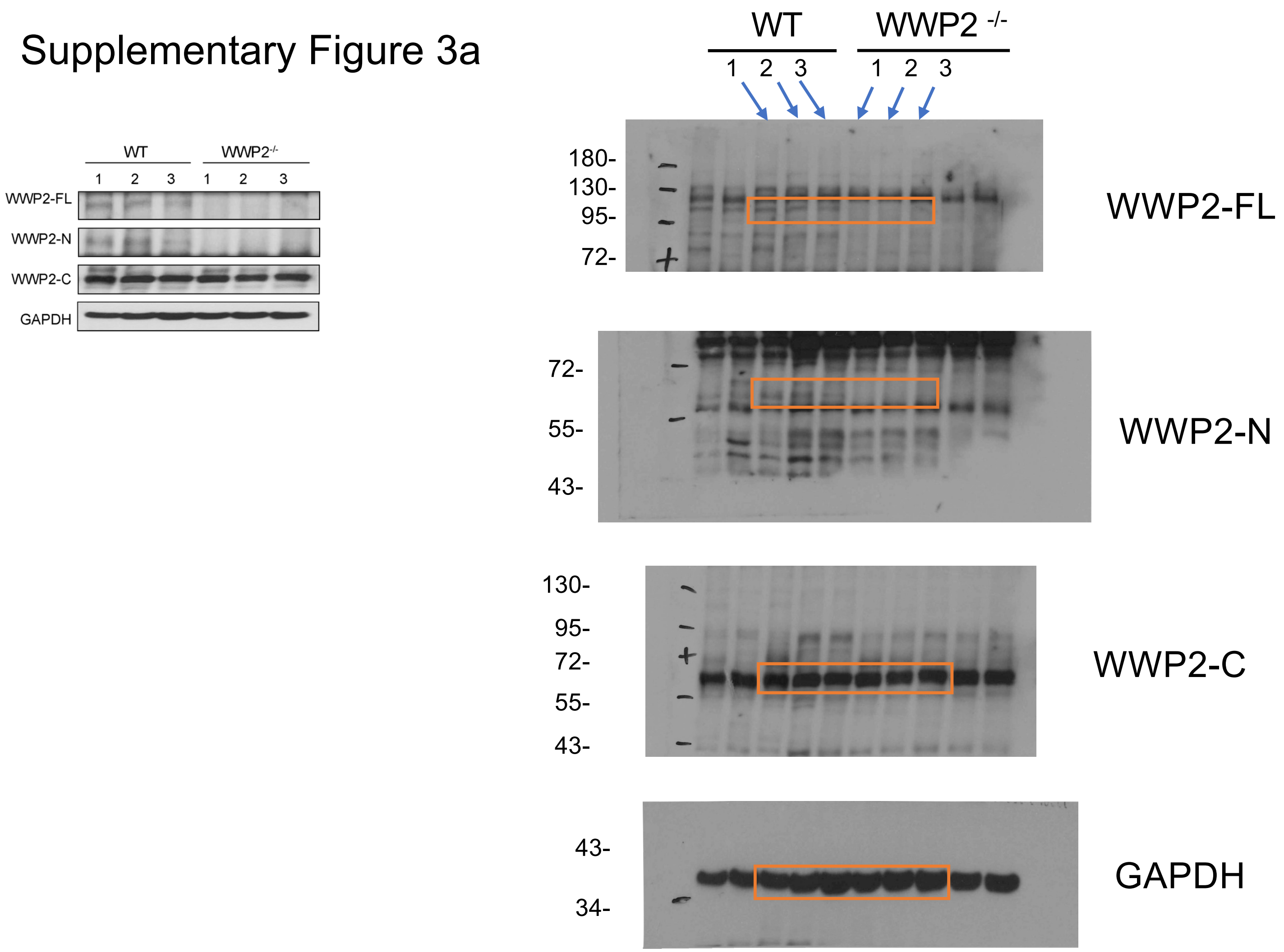

Supplementary Figure 3e

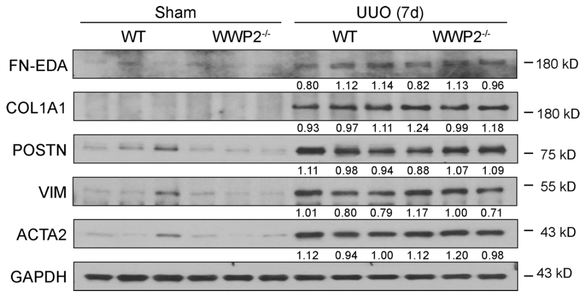

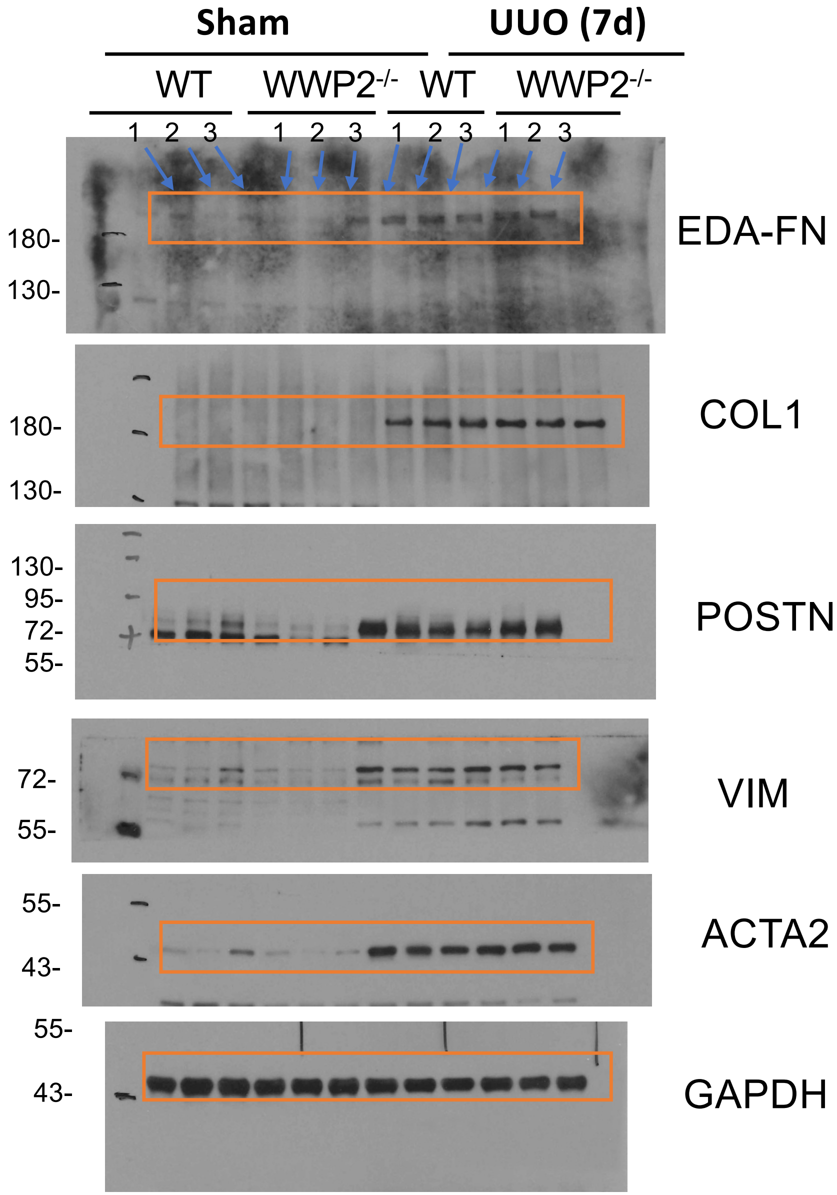

Supplementary Figure 5a

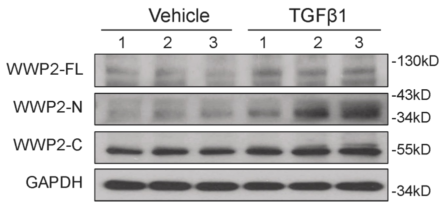

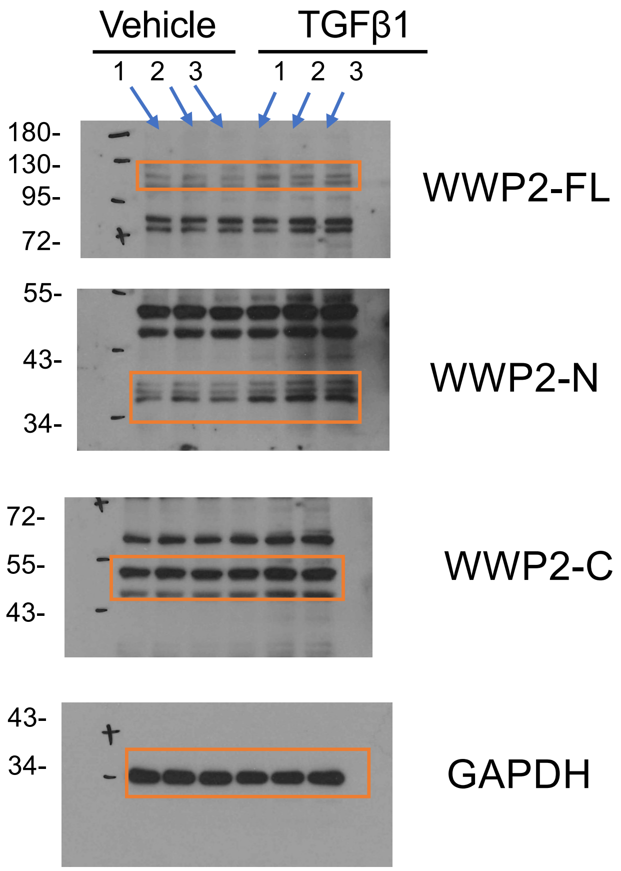

Supplementary Figure 5b

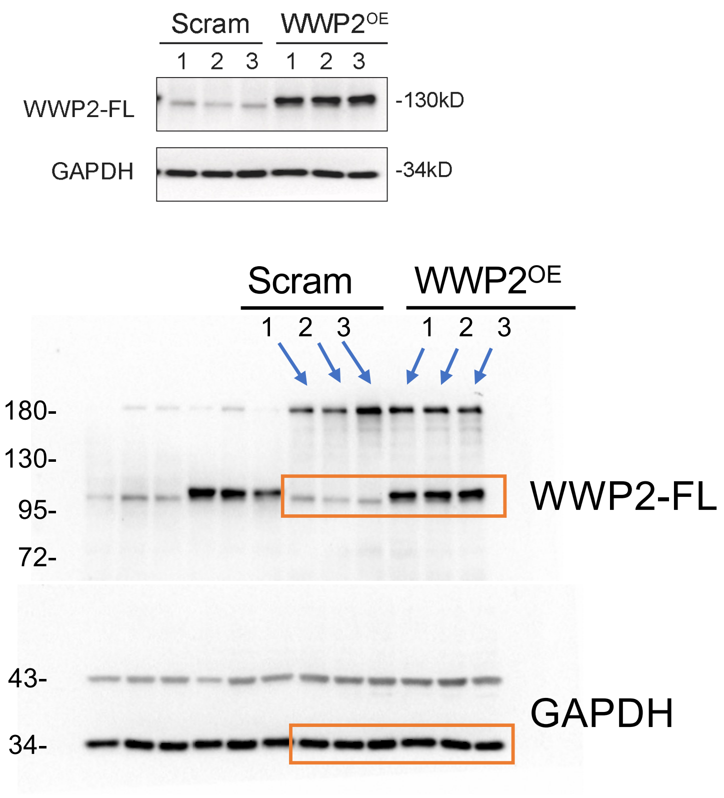

Supplementary Figure 5c

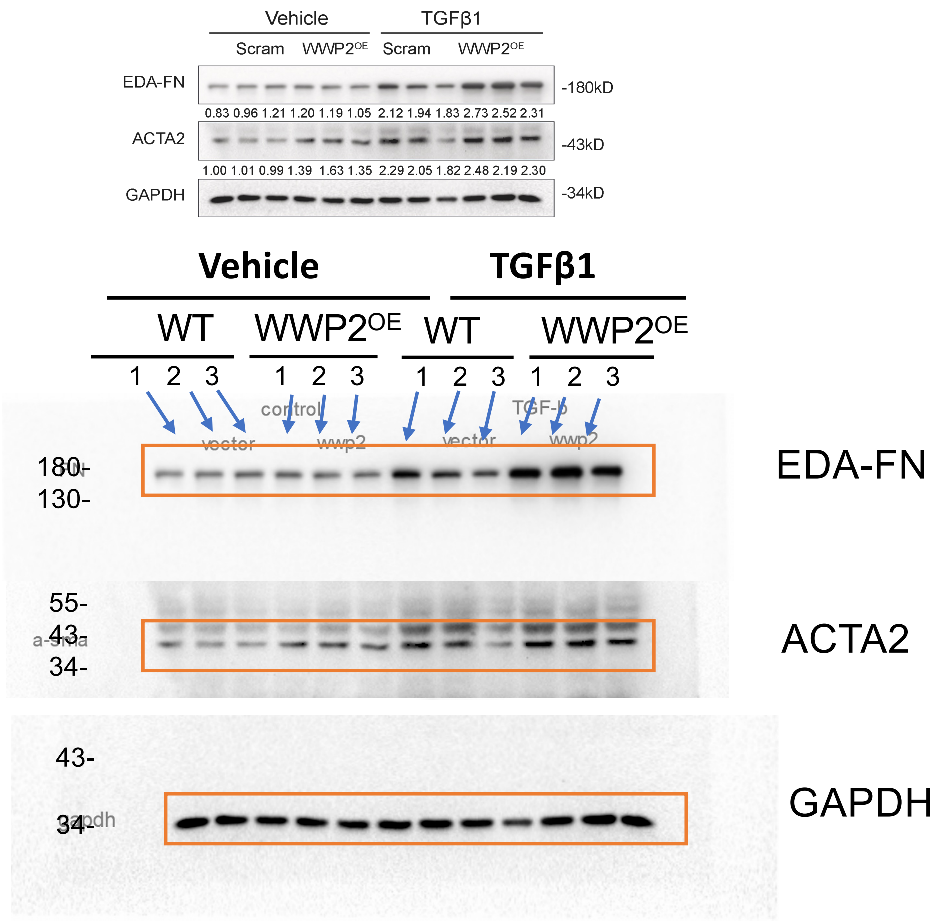

Supplementary Figure 8c

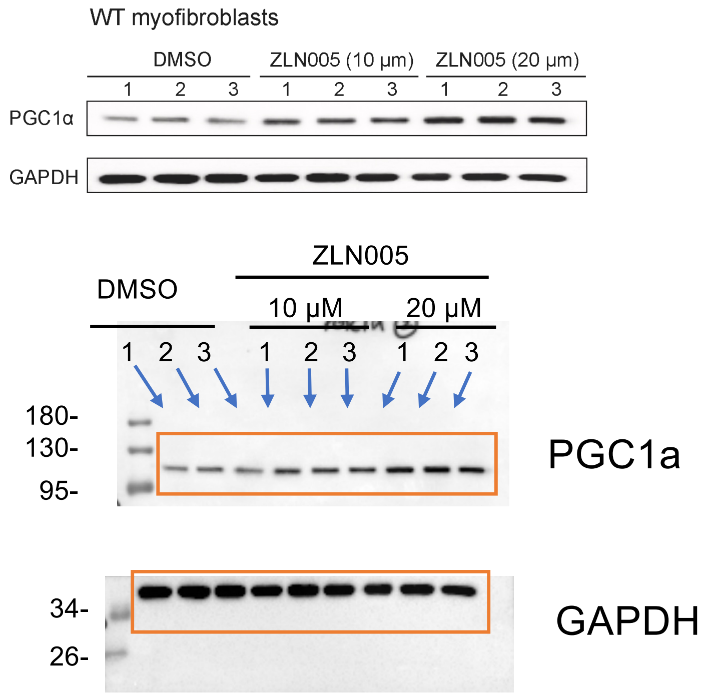

Supplementary Figure 8e

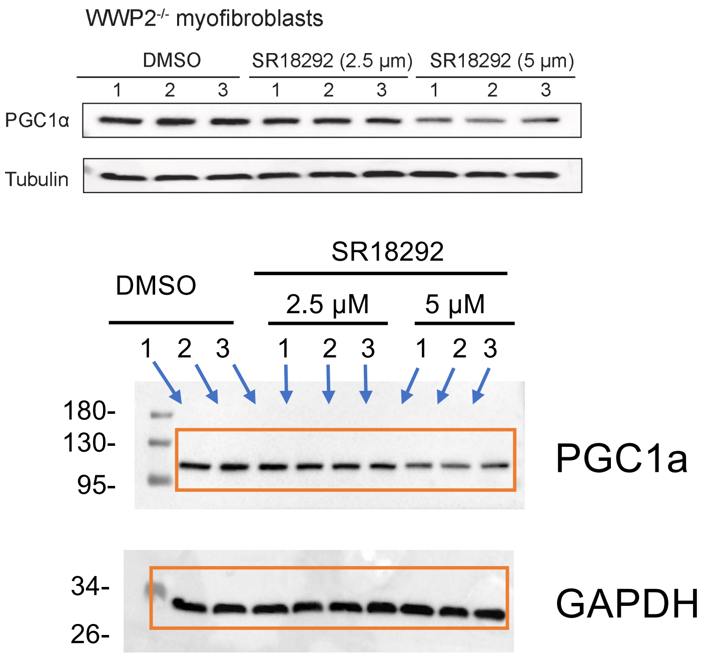

Supplement: Supplementary file 1 [file jasn-35-696-s001.pdf]
